# Synthesis, Biological Evaluation, and Molecular Docking of Pyridine-Containing Squaramides as DNase I Inhibitors

**DOI:** 10.3390/molecules31193548

**Published:** 2026-10-05

**Authors:** Mariyana Atanasova, Nina Ruseva, Georgi Tirolski, Simeon Stoyanov, Ana Marković, Andrija Šmelcerović, Magdalena Angelova, Hristina Sbirkova-Dimitrova, Adriana Bakalova, Rositsa Mihaylova, Emiliya Cherneva

**Affiliations:** 1 Department of Chemistry, Faculty of Pharmacy, Medical University of Sofia, 2 Dunav Str., 1000 Sofia, Bulgaria; a_bakalova@pharmfac.mu-sofia.bg; 2 Centre of Excellence in Informatics and Information and Communication Technologies, Bulgarian Academy of Sciences, Akad. Georgi Bonchev Str., Block 2 and 25A, 1113 Sofia, Bulgaria; rmihaylova@pharmfac.mu-sofia.bg; 3 Department of General and Inorganic Chemistry, University of Chemical Technology and Metallurgy, 1756 Sofia, Bulgaria; nina.ruseva@uctm.edu; 4 Institute of Organic Chemistry with Centre of Phytochemistry, Bulgarian Academy of Sciences, Acad. G. Bontchev Str., Bl. 9, 1113 Sofia, Bulgaria; g_tirolski@abv.bg (G.T.); Simeon.Stoyanov@orgchm.bas.bg (S.S.); 5 Department of Pharmacology, Pharmacotherapy and Toxicology, Faculty of Pharmacy, Medical University of Sofia, 2 Dunav Str., 1000 Sofia, Bulgaria; 6 Department of Pharmacy, Faculty of Medicine, University of Niš, Blvd. Dr Zorana Đinđića 81, 18000 Niš, Serbia; anule89@yahoo.com; 7 Department of Chemistry, Faculty of Medicine, University of Niš, Blvd. Dr Zorana Đinđića 81, 18000 Niš, Serbia; a.smelcerovic@yahoo.com; 8 Institute of Mineralogy and Crystallography, “Akad. Ivan Kostov”, Bulgarian Academy of Sciences, Acad. Georgi Bonchev Str., Bl. 107, 1113 Sofia, Bulgaria; magdalena.angelova@imc.bas.bg (M.A.); hsd@imc.bas.bg (H.S.-D.); 9 Center of Competence “Personalized Innovative Medicine”—2 PERIMED—2, Vasil Aprilov Blvd. 15A, 4002 Plovdiv, Bulgaria

**Keywords:** squaramides, pyridine-containing compounds, DNase I inhibitors, enzyme inhibition, molecular docking

## Abstract

Background: DNase I inhibitors may be useful as biochemical tools and as potential leads for investigating pathological processes associated with excessive DNA degradation. Squaramides represent attractive scaffolds for drug discovery owing to their rigid, highly polarized structure and versatile hydrogen-bonding properties. Methods: Four novel pyridine-containing squaramides (**5a**–**5d**), comprising two symmetric and two asymmetric derivatives, were synthesized and structurally characterized by spectroscopic methods and, for **5a** and **5b**, single-crystal X-ray diffraction. Their DNase I inhibitory activity and cytotoxicity were evaluated experimentally. Comparative structure–activity analysis, molecular docking, molecular descriptor calculations, and in silico ADME profiling were additionally performed. Results: All compounds inhibited bovine pancreatic DNase I, with IC_50_ values ranging from 34.42 ± 5.86 to 61.95 ± 9.61 μM. Compound **5a** showed the highest inhibitory activity within the present series (IC_50_ = 34.42 ± 5.86 μM). Comparison with five previously reported structurally related derivatives identified preliminary structure–activity trends, with **5a** exhibiting the highest activity among the nine compounds considered. Molecular docking predicted a common preferred binding region within the major DNA-contact/catalytic region of DNase I and suggested possible noncovalent ligand–enzyme interactions. Molecular descriptor calculations revealed differences in the electronic properties of selected compounds, while in silico ADME analysis predicted generally favorable physicochemical and absorption-related properties together with potential pharmacokinetic liabilities. No measurable cytotoxicity was observed against the investigated cancer cell lines at the tested concentrations. Conclusions: Pyridine-containing squaramides represent a promising scaffold for further investigation as DNase I inhibitors, with **5a** providing a useful starting point for subsequent structural optimization and mechanistic studies.

## 1. Introduction

Deoxyribonuclease I (DNase I) is a well-characterized mammalian endonuclease that catalyzes the hydrolysis of phosphodiester bonds in single- and double-stranded DNA, generating 3′-hydroxyl and 5′-phosphate termini [1,2]. The enzyme is a secreted glycoprotein [3] found predominantly in the pancreas, blood, and other body fluids, where it contributes to the degradation and clearance of extracellular DNA released during apoptosis, necrosis, and neutrophil extracellular trap formation (NETosis) [4,5,6,7,8]. The enzyme requires Mg^2+^ and Ca^2+^ ions and exhibits optimal activity at a neutral pH [8,9]. Beyond its established role in extracellular DNA clearance, DNase I has also been implicated in cellular processes associated with apoptosis and Fas receptor regulation [8,10,11]. Altered DNase I activity has been reported in a range of pathological conditions, including neurodegenerative and autoimmune diseases, graft-versus-host disease, ischemic injury, type 2 diabetes, idiopathic dilated cardiomyopathy, drug-induced tissue injury, and radiation-induced damage [2,12,13,14,15,16,17,18,19,20,21,22,23,24,25]. These observations have stimulated interest in the pharmacological modulation of DNase I activity.

DNase I inhibitors may have potential applications not only in the investigation of pathological processes associated with excessive DNA degradation but also as biochemical and diagnostic tools. For example, endogenous inhibitors of pancreatic DNase I have been investigated as potential indicators of pancreatic inflammation and early markers of chronic pancreatic disorders [26]. Despite these potential applications, the development of DNase I inhibitors remains relatively limited, and no clinically validated “gold-standard” small-molecule inhibitor has yet been established.

A variety of structurally diverse small organic DNase I inhibitors have been reported in recent years. These include benzimidazole derivatives, among which the most active reported compound exhibited an IC_50_ value of 79.46 ± 11.75 μM [27], 4-thiazolidinone derivatives with IC_50_ values in the low-hundred-micromolar range, including a representative compound with an IC_50_ of 115.96 ± 11.70 μM [28], and 5,6,7,8-tetrahydrobenzo[4,5]thieno[2,3-d]pyrimidines, for which IC_50_ values ranging from 106 μM to >200 μM have been reported [29]. Other investigated scaffolds include benzocyclobutane-2,5-diones [30], rutin, which inhibited bovine pancreatic DNase I with an IC_50_ of 108.90 ± 9.73 μM [31], 4-(4-chlorophenyl)thiazol-2-amines [12], 1-(pyrrolidin-2-yl)propan-2-one derivatives [32], 1,2,3,4-tetrahydroisoquinoline derivatives [33], and 4-oxothiazolidines, for which the most active derivative exhibited an IC_50_ of 67.94 ± 5.99 μM [34]. More recently, halogenated chalcones [35] and thiazole derivatives [36] have further expanded the structural diversity of DNase I inhibitors.

Particularly notable activity has been reported for 8-hydroxyquinoline-based compounds. Three derivatives described by Gajić et al. exhibited IC_50_ values of 23.33, 25.61, and 30.58 μM, respectively, representing some of the most potent synthetic small-molecule DNase I inhibitors reported to date [37]. These findings highlight the potential of appropriately substituted heteroaromatic scaffolds for achieving sub-50 μM DNase I inhibition.

Our previous studies have also identified pyridine-containing squaramides as promising DNase I inhibitors. The most active monosquaramide, **3c**, exhibited an IC_50_ of 48.04 ± 7.98 μM [38], while a subsequently investigated chloro-substituted pyridine squaramate showed improved activity, with an IC_50_ of 43.82 ± 6.51 μM [39]. Collectively, these findings indicate that squaramide-containing scaffolds can provide a favorable structural framework for DNase I inhibition.

Nevertheless, despite the increasing number of reported small-molecule DNase I inhibitors, the available compounds encompass structurally diversed chemotypes, and systematic information on the structural features governing their inhibitory activity remains limited. Further exploration of structurally related series is therefore needed to identify molecular features associated with DNase I inhibition and to provide a basis for the rational design of new inhibitors.

Squaramides are nitrogen-containing derivatives of squaric acid characterized by a cyclobutenedione core bearing two NH hydrogen-bond donors and two carbonyl oxygen acceptors. Their rigid and highly polarized structure enables diverse hydrogen-bonding interactions, making squaramides attractive scaffolds for molecular recognition and drug discovery [40,41,42,43]. Accordingly, squaramide-containing compounds have been investigated for a broad range of biological activities, including antiparasitic, antibacterial, antiviral, and anticancer effects [44,45,46,47,48,49,50]. The pharmaceutical relevance of this scaffold is further supported by the development of several biologically active squaramide-containing compounds, including navarixin (MK-7123) and perzinfotel (EAA-090), which have reached clinical evaluation [40,51,52,53,54].

Our previous studies demonstrated that pyridine-containing squaramates and squaramides can inhibit DNase I, identifying this class of compounds as a suitable starting point for further structural investigation [38,39]. However, the compounds investigated previously were predominantly monosubstituted squaric acid derivatives, and the influence of extending this framework to disubstituted squaramides has not been systematically explored. In particular, it remains unclear how changes in the overall molecular architecture, including molecular symmetry and the nature and arrangement of the pyridine-containing substituents around the squaramide core, affect DNase I inhibitory activity. Addressing these questions may provide further insight into the structural requirements associated with DNase I inhibition and support the rational design of additional analogues.

Motivated by these considerations, the present study extends our previous work from monosubstituted pyridine-containing squaramides and squaramates to a series of disubstituted squaramides. Four compounds (**5a**–**5d**), comprising two and two asymmetric derivatives, were designed and synthesized to explore how variations in molecular symmetry and pyridine-containing substituents are associated with DNase I inhibitory activity. The compounds were structurally characterized by elemental analysis and spectroscopic methods, while the structures of the symmetric derivatives **5a** and **5b** were additionally confirmed by single-crystal X-ray diffraction. Their DNase I inhibitory activity was evaluated experimentally and considered in the context of structurally related compounds previously reported by our group to identify preliminary structure–activity trends. Cytotoxicity against selected human cancer cell lines was also investigated as a preliminary assessment of their biological profile. Molecular docking and in silico ADME profiling were employed as complementary approaches to explore possible ligand–enzyme interactions and predicted pharmacokinetic properties, respectively.

## 2. Results and Discussion

### 2.1. Chemistry

A series of four pyridine-containing squaramides (**5a**–**5d**), comprising two symmetric derivatives (**5a** and **5b**) and two asymmetric derivatives (**5c** and **5d**), was synthesized to investigate the influence of molecular symmetry and pyridine substitution on DNase I inhibitory activity. The synthetic strategy was based on the well-established nucleophilic substitution of diethyl squarate by primary amines, which enables the preparation of mono- and disubstituted squaramides by controlling the reaction stoichiometry and sequence [55].

**Scheme 1:**
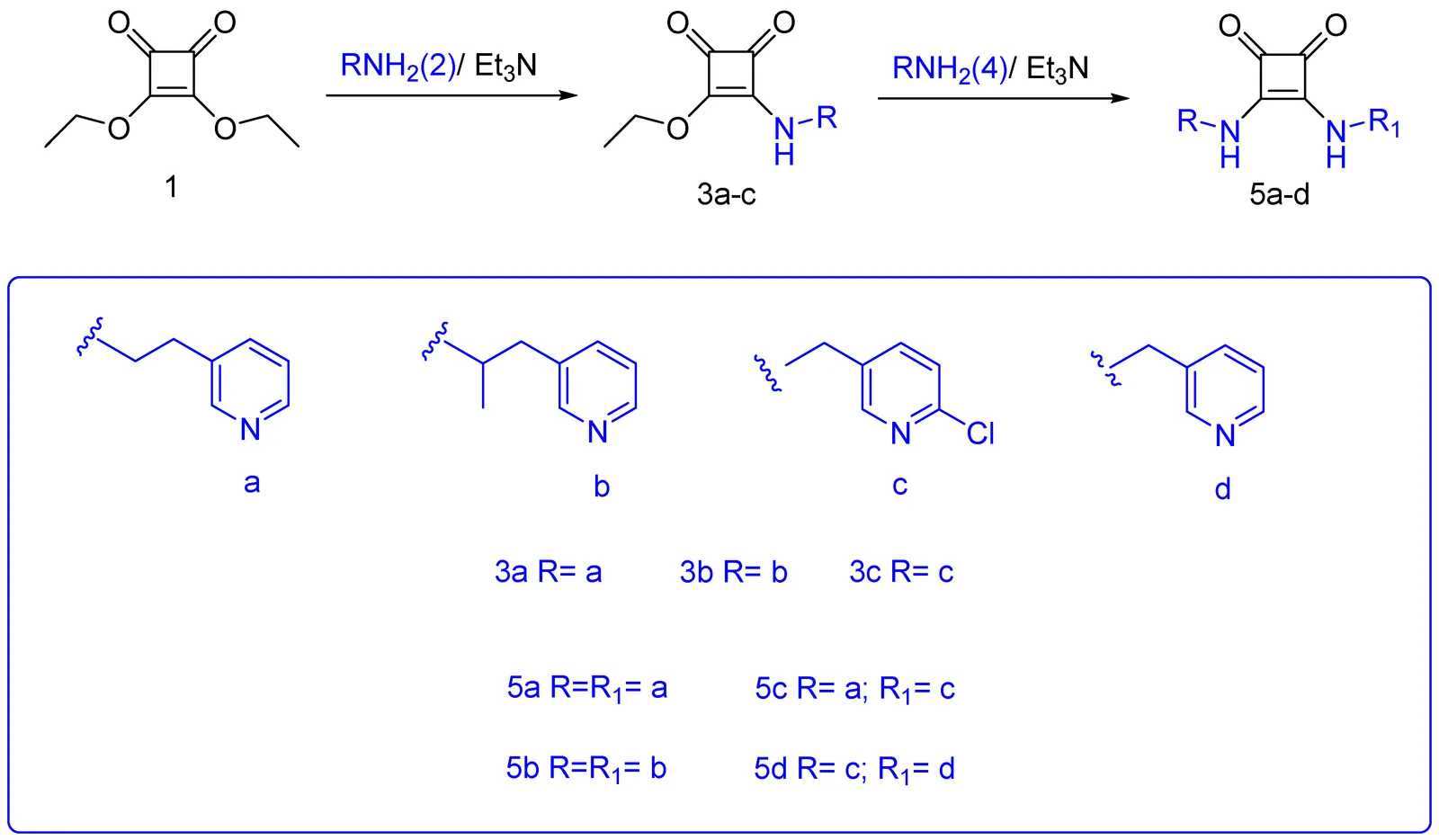
Synthetic routes to symmetric (**5a**, **5b**) and asymmetric (**5c**, **5d**) pyridine-containing squaramides.

The asymmetric derivatives **5c** and **5d** were prepared through a two-step procedure. In the first step, diethyl squarate was reacted with the appropriate pyridine-containing amine to afford the corresponding monosubstituted squaramate intermediate. Subsequent reaction with a second amine provided the desired asymmetric squaramides. In contrast, the symmetric derivatives **5a** and **5b** were obtained by direct reaction of diethyl squarate OR diethyl ester of squaric acid with two equivalents of the corresponding amine, providing an efficient one-step route to the disubstituted products. The overall synthetic pathway is summarized in Scheme 1.

The identity and purity of compounds **5a**–**5d** were confirmed by elemental analysis, FT-IR, and^1^H and ^13^C NMR spectroscopy. In addition, the molecular structures of the symmetric derivatives **5a** and **5b** were unambiguously established by single-crystal X-ray diffraction. The corresponding crystallographic data and selected structural parameters are provided in the Supporting Information.

### 2.2. Spectroscopic Analysis

The synthesized squaramides **5a**–**5d** were characterized by FT-IR, and^1^H NMR, and ^13^C NMR spectroscopy. The spectroscopic data were consistent with the proposed molecular structures.

In the FT-IR spectra, all four compounds exhibited N–H stretching vibrations in the 3186–3153 cm^−1^ range, consistent with the presence of the squaramide NH groups. Compared with the corresponding squaramates reported previously [38,39], the asymmetric C=O stretching bands of the squaramides were shifted to lower wavenumbers by approximately 40–50 cm^−1^ and appeared as medium-intensity bands in the 1644–1638 cm^−1^ range. The symmetric C=O stretching vibrations were observed at approximately 1790 cm^−1^. A strong band at approximately 1540 cm^−1^ was assigned to the C=C stretching vibration of the cyclobutenedione ring. This band was relatively broad, which may be attributed to overlap with vibrations associated with the pyridine moieties. The N–H deformation vibrations were observed in the 1480–1475 cm^−1^ range. Collectively, these characteristic absorptions support the formation of the squaramide functionality and are consistent with its conjugated structure and hydrogen-bonding capacity.

The ^1^H NMR showed resonances attributable to the squaramide NH protons in the 8.87–8.42 ppm range. Signals corresponding to the pyridine protons were observed in their expected chemical-shift regions, depending on the nature of the linker connecting the pyridine ring to the squaramide moiety. The ^13^C-NMR further supported the proposed structures. Signals assigned to the carbon atoms of the cyclobutenedione ring were observed in the 189–167 ppm range. In contrast to the previously investigated squaramates [38,39], no duplication of the carbon signals was observed for the synthesized squaramides. The remaining aromatic and aliphatic carbon resonances were also consistent with the proposed structures.

The spectroscopic data were consistent with the proposed structures of **5a**–**5d**, while the molecular structures of **5a** and **5b** were further confirmed by single-crystal X-ray diffraction.

### 2.3. Crystal Structure Analysis

The molecular structures of the symmetric squaramides **5a** and **5b** were confirmed by single-crystal X-ray diffraction. Their crystallographic features were compared with those of the previously reported squaramates **3a** and **3c** [38,39]. Compounds **5a** and **5b** crystallize in non-centrosymmetric orthorhombic space groups, Pna2_1_ and Pca2_1_, respectively, whereas the corresponding squaramates **3a** and **3c** crystallize in centrosymmetric space groups [38,39]. The asymmetric units of both **5a** and **5b** contain one independent molecule, and the corresponding unit cells contain four molecules (Table S1, Supporting Information). The refinement of the crystal structures of **5a** and **5b** was associated with some residual electron density and non-ideal ADP values for several atoms, as also reflected by the checkCIF alerts. These features are related to the quality of the diffraction data and refinement; however, the molecular connectivity and overall structures of both compounds are unambiguous.

**Figure 1:**
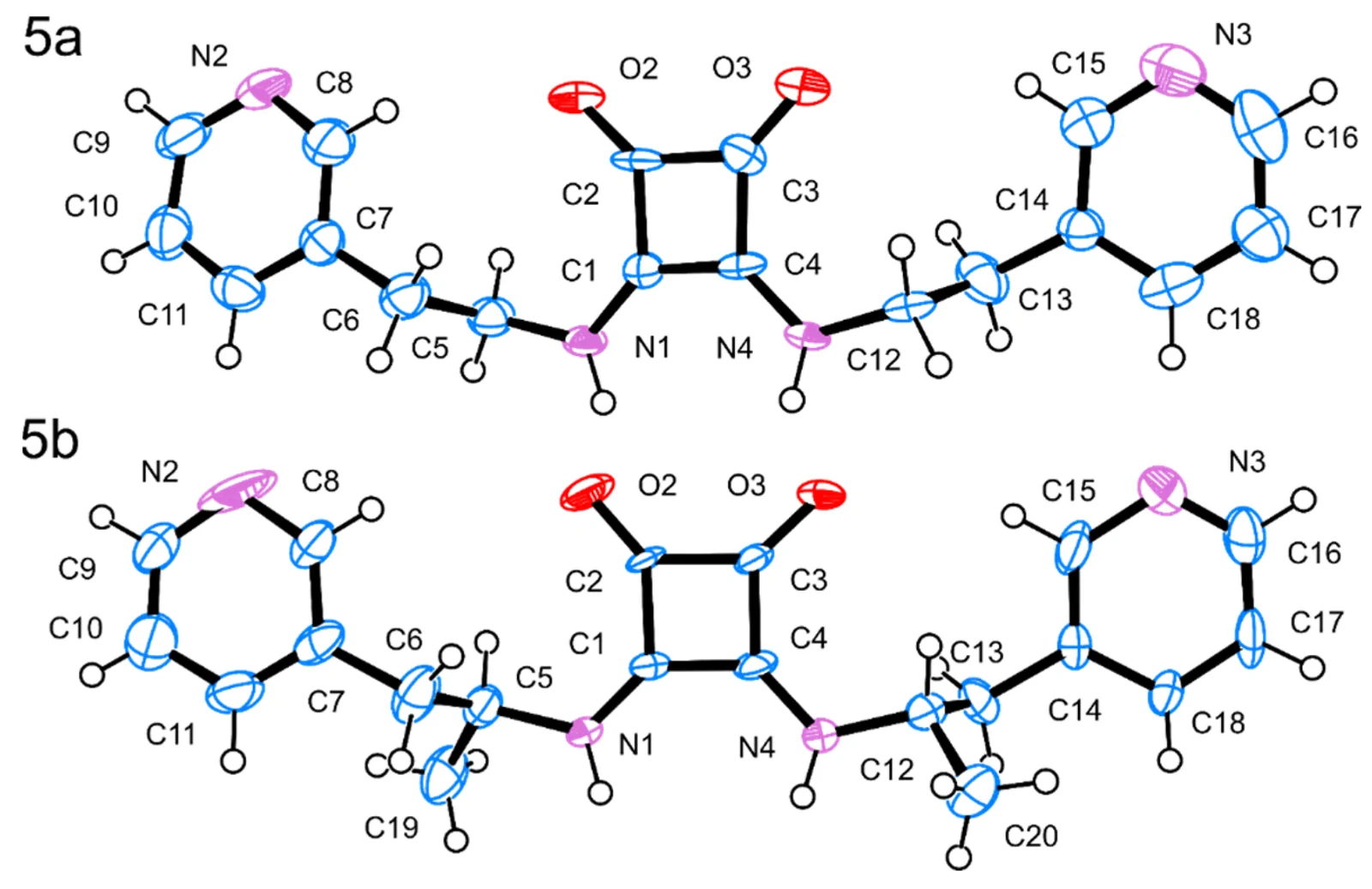
ORTEP representations of the molecular structures of **5a** and **5b**, with thermal ellipsoids drawn at the 50% probability level.

The molecular structures of **5a** and **5b** are shown in Figure 1. Both compounds consist of three aromatic fragments connected through ethylamine linkers to the central squaramide moiety. The aromatic fragments adopt approximately parallel orientations. The pyridine rings at both ends of the molecules are highly planar, with root-mean-square deviations (rmsd) of 0.003 and 0.004 Å for **5a** and **5b**, respectively, while the central squaramide fragment shows an rmsd of 0.044 Å.

The conformations of the ethylamine linkers in **5a** and **5b** are reflected by the N1–C5–C6–C7 and N4–C12–C13–C14 torsion angles. The N1–C5–C6–C7 torsion angles are 162.1° and 164.9° for **5a** and **5b**, respectively, whereas the corresponding N4–C12–C13–C14 torsion angles are −169.3° and 161.6°. Thus, both compounds adopt largely extended conformations along these linker segments, although differences in their spatial arrangement are evident from the molecular structures shown in Figure 1.

**Figure 2:**
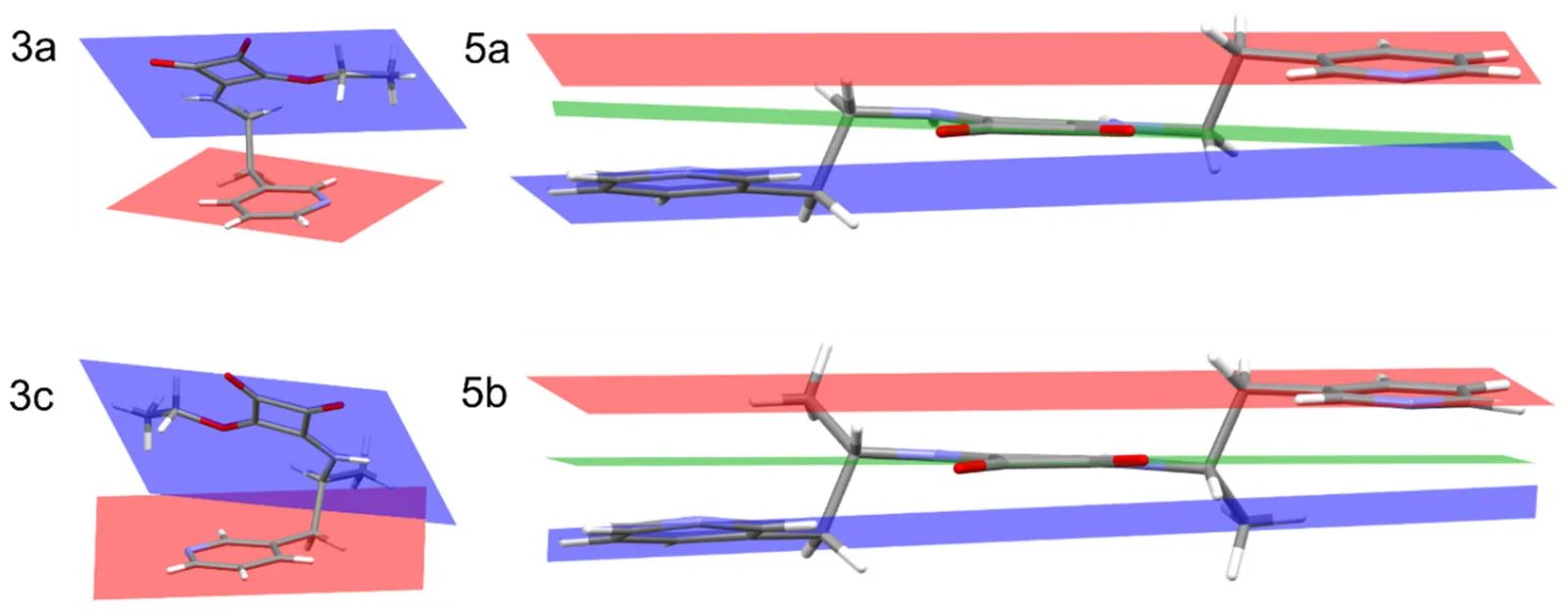
Comparison of the molecular structures of squaramides **5a** and **5b** and the corresponding squaramates **3a** and **3c**.

The bond lengths within the central squaramide fragment also reveal differences between **5a** and **5b**. For **5a** and **5b**, respectively, the C1–C2 bond lengths are 1.469 and 1.450 Å, C2–C3 are 1.496 and 1.489 Å, C1–C4 are 1.427 and 1.449 Å, and C3–C4 are 1.451 and 1.438 Å. The corresponding carbonyl C2–O2 and C3–O3 bond lengths are 1.227 and 1.216 Å in **5a** and 1.220 and 1.259 Å in **5b**, respectively. The variation among the C–C and C=O bond lengths indicates an asymmetric distribution of bond lengths within the formally conjugated squaramide core, particularly in **5b**. Compared with the previously reported squaramates **3a** and **3c** [38,39], these structural differences are consistent with modification of the squaric-acid-derived core upon replacement of the ester functionality by the second NH-substituted group in the squaramide derivatives. In **3a** and **3c**, the pyridine rings are essentially coplanar with the squaramate fragment, with rmsd values of 0.001–0.002 Å [38,39]. A comparison of the molecular geometries of **3a**, **3c**, **5a**, and **5b** is presented in Figure 2.

The molecules are linked predominantly by intermolecular N–H···O hydrogen bonds involving the squaramide carbonyl groups. Additional weak C–H···N and C–H···O interactions were observed in the crystal of **5a**, whereas weak C–H···O interactions involving methyl groups were identified in **5b** (Table 1 and Table 2). Despite the presence of three aromatic fragments, no significant π···π stacking interactions were observed.

**Figure 3:**
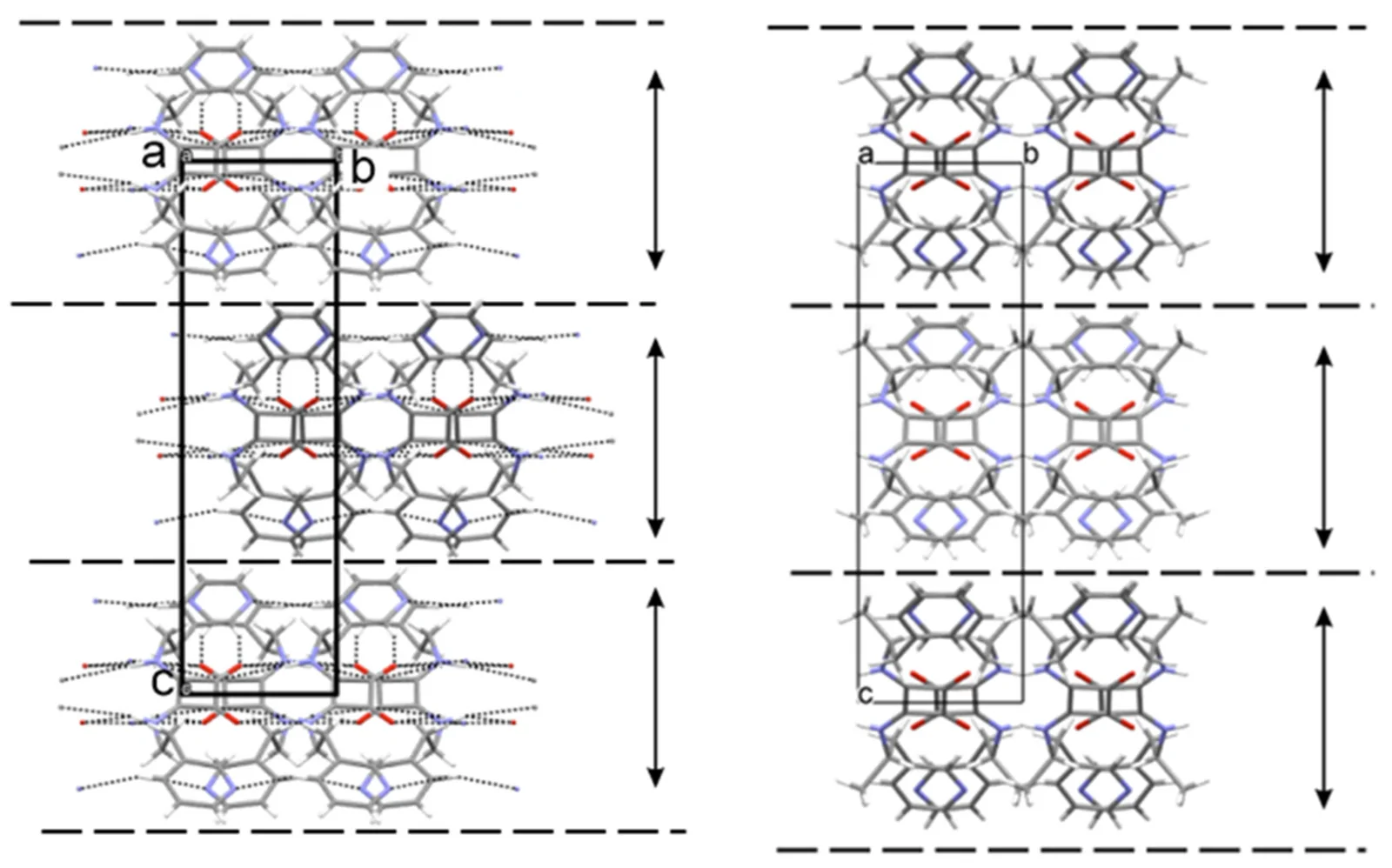
Pseudo-layered crystal packing of squaramides **5a** and **5b**. The double-headed arrows indicate the extent of the repeating pseudo-layers.

The intermolecular hydrogen-bonding interactions result in the formation of pseudo-layered packing arrangements in both crystals. The squaramide groups and their associated hydrogen-bonding interactions are located predominantly within the layers, while the pyridine rings are oriented toward the outer regions (Figure 3).

### 2.4. Quantum Chemical Calculations

Density functional theory (DFT) calculations were performed to optimize the molecular geometries of the synthesized squaramides and to assess their agreement with the experimentally determined crystal structures (Figure S1). Geometry optimizations were carried out using the B3LYP functional with the 6-311++G(d) basis set [56,57,58,59]. Selected bond lengths, valence angles, and dihedral angles obtained from the optimized structures were compared with the corresponding experimental parameters determined by single-crystal X-ray diffraction for compounds **5a** and **5b**. The optimized structures, calculated geometric parameters for compounds **5a**–**5d**, and atom numbering are provided in the Supporting Information (Table S2 and Scheme S1).

The calculations showed that the squaramide moiety retained an essentially planar geometry after optimization, consistent with significant delocalization within the cyclobutenedione ring. The calculated geometric parameters showed good agreement with the corresponding experimental data for compounds **5a** and **5b**, as well as with previously reported structural data for squaramates **3a**, **3c**, **3d**, and **3e** [38,39]. The small differences between calculated and experimental parameters may arise from crystal-packing effects and intermolecular interactions, particularly hydrogen bonding, which are not represented in the isolated-molecule calculations.

### 2.5. In Silico ADME Analysis

The physicochemical, drug-likeness, and pharmacokinetic properties of compounds **5a**–**5d** were evaluated using the SwissADME web platform (available on https://www.swissadme.ch/index.php, accessed on 9 March 2026). The calculated parameters are summarized in Table 3.

The molecular weights of compounds **5a**–**5d** ranged from 322.36 to 350.41 g/mol, which are compatible with molecular sizes generally encountered in lead-like and drug-like chemical space [60]. According to the drug-likeness criteria implemented in SwissADME, all four compounds were classified as drug-like. In contrast, the lead-likeness criteria were fulfilled only by **5c** and **5d**. Compound **5a** showed one lead-likeness violation due to having more than seven rotatable bonds, whereas **5b** showed two violations, namely molecular weight above 350 g/mol and more than seven rotatable bonds.

The consensus logP values ranged from 1.35 to 1.97, indicating moderate lipophilicity. Compounds **5a** and **5d** showed the lowest values (1.35 and 1.41, respectively), suggesting a relatively balanced hydrophilic–lipophilic profile, whereas compounds **5b** and **5c** exhibited somewhat higher values (1.64 and 1.97, respectively), while remaining within a moderate lipophilicity range. Such a lipophilicity profile may provide a favorable balance between aqueous compatibility and the ability to partition into hydrophobic environments, which can support both dissolution and passive membrane permeation. The differences in lipophilicity across the series can be rationalized in relation to their structural modifications. Compounds **5a** and **5b** differ by an additional methyl group in each substituent of **5b**, corresponding to two additional methyl groups overall. This modification is accompanied by an increase in consensus logP of approximately 0.29 units (from 1.35 for **5a** to 1.97 for **5b**). Compound **5d** contains alkyl chains shortened by one methylene group relative to **5a**, together with a chlorine substituent on one pyridine ring. Despite the reduction in the alkyl-chain length, its logP value (1.41) remains close to that of **5a**, suggesting that the chlorine substitution contributes to maintaining the overall lipophilicity. In compound **5c**, one substituent contains an additional methylene group relative to **5d**, resulting in a modest increase in logP from 1.41 to 1.64. Overall, these observations indicate that the calculated lipophilicity reflects the combined effects of alkyl-chain length and pyridine substitution within this series.

All four compounds possessed four hydrogen-bond acceptors and two hydrogen-bond donors. These values are consistent with the hydrogen-bonding criteria of Lipinski’s Rule of Five. In addition, all four compounds were classified as drug-like according to the combined drug-likeness criteria implemented in SwissADME, including Lipinski, Ghose, Veber, Egan, and Muegge filters.

The number of rotatable bonds ranged from six to eight. Compounds **5a** and **5b** contained eight rotatable bonds, whereas **5c** and **5d** contained seven and six, respectively. Thus, the chlorinated derivatives, particularly **5d**, were predicted to exhibit somewhat lower conformational flexibility. The higher number of rotatable bonds in **5a** and **5b** accounts for their respective lead-likeness violations.

The topological polar surface area (TPSA) was identical for all four compounds (83.98 Å^2^), reflecting their closely related hydrogen-bonding characteristics. This value is below the commonly applied 140 Å^2^ threshold associated with favorable gastrointestinal absorption, while being above the range generally associated with efficient passive blood–brain barrier penetration [61,62,63,64]. Consistent with this, all four compounds were predicted to have high gastrointestinal absorption but were classified as non-BBB-permeantion.

The predicted ESOL (Estimated SOLubility model [65]) logS values ranged from −2.80 to −3.48, corresponding to estimated aqueous solubilities of 3.29 × 10^−4^ to 1.60 × 10^−3^ mol/L. All four compounds were classified as soluble, with **5a** showing the highest predicted solubility and **5b** the lowest, consistent with its higher molecular weight and lipophilicity compared with **5a**.

All four compounds were predicted to have high gastrointestinal absorption. The predicted oral bioavailability category was reported as unsaturated (INSATU) for **5a**, **5c**, and **5d**, whereas **5b** was classified as saturated (SATU). The bioavailability score was 0.55 for all four compounds. This computational score should not be interpreted as a direct prediction of 55% oral bioavailability, but rather as a SwissADME-derived estimate of the likelihood of achieving favorable bioavailability.

None of the compounds was predicted to permeate the blood–brain barrier, which is consistent with their TPSA values and suggests limited passive penetration into the central nervous system. Regarding P-glycoprotein, compounds **5a** and **5b** were predicted to be P-gp substrates, whereas **5c** and **5d** were not. This difference may contribute to differences in their predicted disposition and cellular exposure.

The predicted cytochrome P450 inhibition profiles revealed potential metabolic liabilities. All four compounds were predicted to inhibit CYP1A2 and CYP3A4, whereas none was predicted to inhibit CYP2D6. Compounds **5c** and **5d** were additionally predicted to inhibit CYP2C19 and CYP2C9. Compound **5b** was predicted to inhibit CYP2C9, whereas **5a** was not predicted to inhibit either CYP2C19 or CYP2C9. These predictions indicate potential drug–drug interaction liabilities that should be considered in further optimization and experimentally evaluated.

Finally, the predicted synthetic accessibility scores ranged from 2.79 to 3.97. Compounds **5a**, **5c**, and **5d** showed relatively favorable synthetic accessibility scores of 3.01, 2.99, and 2.79, respectively, whereas **5b** had a higher score of 3.97, indicating comparatively greater synthetic difficulty. Nevertheless, all four compounds were obtained through relatively straightforward synthetic procedures.

Overall, the SwissADME analysis indicated generally favorable physicochemical and absorption-related properties for compounds **5a**–**5d**, including drug-like profiles, moderate lipophilicity, predicted high gastrointestinal absorption, and predicted aqueous solubility. At the same time, the predicted P-gp substrate behavior of **5a** and **5b** and the CYP inhibition profiles of the series represent potential pharmacokinetic liabilities that should be addressed in subsequent optimization and experimental studies.

### 2.6. Biological Activity

#### 2.6.1. DNase I Inhibition

The inhibitory activity of squaramides **5a**–**5d** against bovine pancreatic DNase I was evaluated in vitro. Crystal violet was used as a reference inhibitor because no universally established standard DNase I inhibitor is available and to facilitate comparison with previously reported compounds [38,39]. All four synthesized squaramides exhibited DNase I inhibitory activity, with IC_50_ values below 100 μM.

Compound **5a**, a symmetric squaramide bearing a 2-(pyridin-3-yl)ethyl substituent, demonstrated the highest inhibitory activity, with an IC_50_ value of 34.42 ± 5.86 μM. This activity was approximately tenfold higher than that of crystal violet (IC_50_ = 354.72 ± 37.15 μM). The remaining compounds, **5b**, **5c**, and **5d**, exhibited IC_50_ values of 59.61 ± 9.40, 61.95 ± 9.61, and 53.81 ± 8.88 μM, respectively, corresponding to approximately six- to sevenfold greater inhibitory activity than the reference compound.

The inhibitory potency of the synthesized squaramides can be further evaluated in the context of previously reported synthetic small-molecule DNase I inhibitors. Notably, a study by [37] identified several highly potent nitroxoline and 8-hydroxyquinoline derivatives. The nitroxoline derivative N-((1-(benzo[d]thiazol-2-ylmethyl)piperidin-3-yl)methyl)-8-hydroxy-5-nitroquinoline-7-carboxamide exhibited an IC_50_ value of 23.33 μM, while the 8-hydroxyquinoline derivatives N-((1-(2,3-dihydro-1H-inden-2-yl)piperidin-3-yl)methyl)-N-(2-(dimethylamino)ethyl)-8-hydroxyquinoline-2-carboxamide and N-((1-(2,3-dihydro-1H-inden-2-yl)piperidin-3-yl)methyl)-N-(2-(dimethylamino)ethyl)-8-hydroxyquinoline-7-carboxamide showed IC_50_ values of 25.61 and 30.58 μM, respectively [37]. Although these compounds exhibited somewhat higher inhibitory potency than **5a**, the IC_50_ value of 34.42 ± 5.86 μM places **5a** among the most potent synthetic small-molecule DNase I inhibitors reported to date.

Importantly, the present results extend our previous observations for pyridine-containing squaramates and squaramides [38,39]. The most active previously reported pyridine-containing squaramate, **3c**, exhibited an IC_50_ value of 48.04 ± 7.98 μM [38], whereas the present compound **5a** showed a lower IC_50_ value of 34.42 ± 5.86 μM. Importantly, the present results extend our previous observations for pyridine-containing squaramates and squaramides [38,39]. Compound **5a** exhibited higher inhibitory activity (IC_50_ = 34.42 ± 5.86 μM) than the most active previously reported derivatives. To place these findings in a broader structural context, compounds **5a**–**5d** were considered together with the previously investigated analogues in a comparative structure–activity analysis (Section 2.7).

The present inhibition assay was designed to determine inhibitory potency (IC_50_) and does not establish the mechanism of DNase I inhibition. Furthermore, because the compounds were not evaluated against other nucleases, their selectivity for DNase I cannot be determined from the present data. Further kinetic studies performed at different substrate and inhibitor concentrations, together with comparative inhibition studies against other nucleases, will be required to determine the mode of inhibition and establish the nuclease selectivity profile of these compounds.

#### 2.6.2. Cytotoxicity

The cytotoxic activity of compounds **5a**–**5d** was evaluated against four human tumor cell lines: HL-60 (acute promyelocytic leukemia), MCF-7 (hormone-sensitive breast adenocarcinoma), MDA-MB-231 (triple-negative breast adenocarcinoma), and BV-173 (BCR:ABL1-positive leukemia). None of the investigated compounds produced detectable cytotoxic effects under the experimental conditions, with IC_50_ values exceeding 200 μM in all tested cell lines.

These findings are particularly relevant when considered alongside the DNase I inhibitory activity of the compounds. While **5a**–**5d** inhibited bovine pancreatic DNase I with IC_50_ values ranging from 34.42 to 61.95 μM, no appreciable reduction in tumor-cell viability was observed even at substantially higher concentrations. This distinction is relevant because pancreatic DNase I is a secreted endonuclease, and inhibition measured against the isolated enzyme does not imply inhibition of an intracellular target or predict a direct cytotoxic response. Nevertheless, given the reported involvement of DNase I in apopto-sis-related processes, assessment of cellular viability provides complementary information on the biological profile of these compounds. The absence of detectable cytotoxicity at concentrations several-fold higher than those required for DNase I inhibition therefore indicates that, under the present experimental conditions, DNase I inhibitory activity was not accompanied by measurable cytotoxicity in the investigated cell lines. However, these findings are limited to the four tumor cell lines examined and should not be interpreted as evidence of DNase I selectivity, a broad safety profile, or therapeutic potential. Further studies in non-tumor cell models, together with comparative evaluation against other nucleases and additional biological and toxicological investigations, will be required to characterize the selectivity and safety profile of these compounds.

### 2.7. Comparative Structure–Activity Analysis

**Figure 4:**
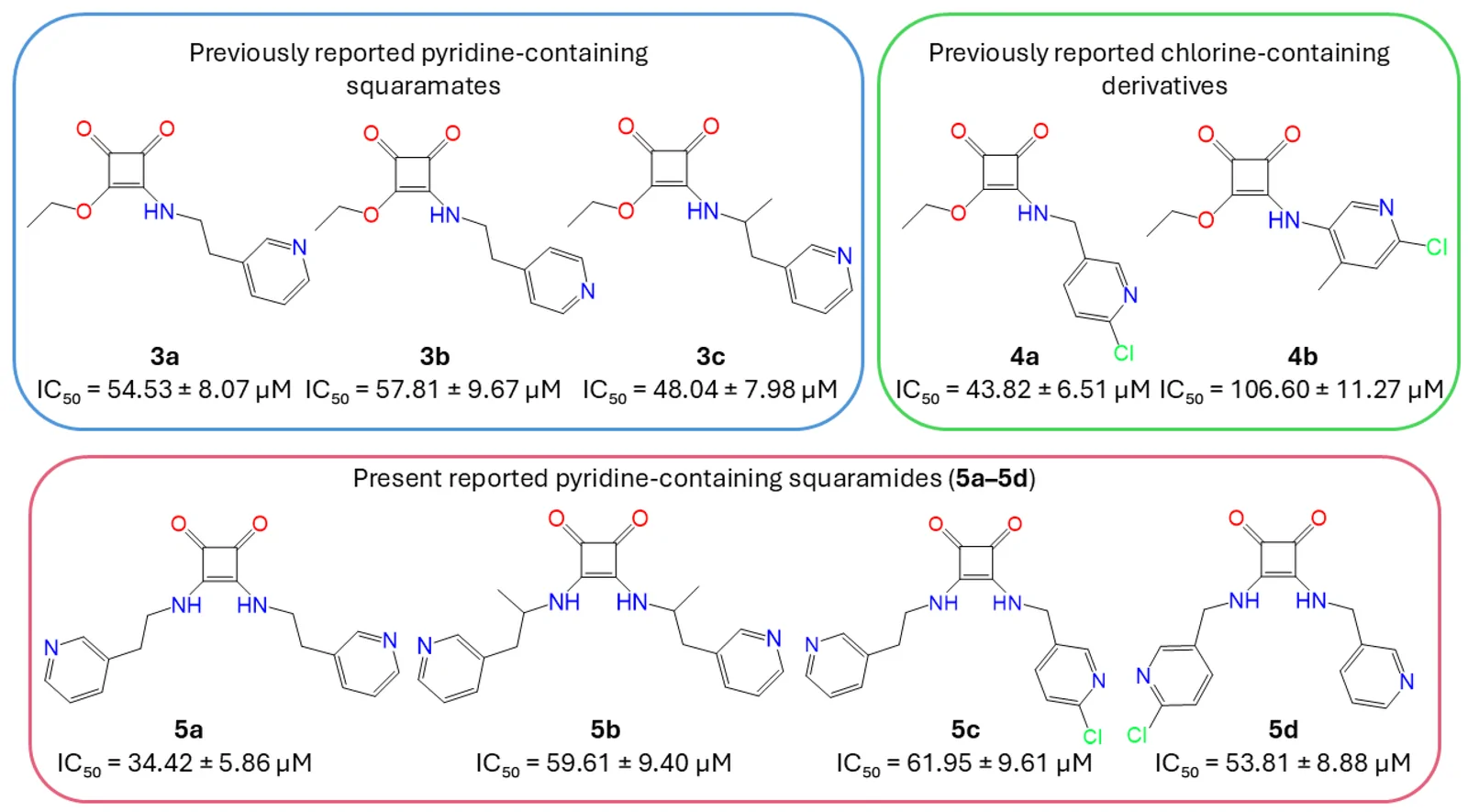
Structures and DNase I inhibitory activity (IC_50_ values) of the nine squaramides and squarametes.

To obtain a broader perspective on the structural features associated with DNase I inhibition, the activities of compounds **5a**–**5d** were compared with those of five structurally related pyridine-containing squaramides and squaramates previously reported by our group [38,39]. Although the resulting set of nine compounds remains relatively small and several structural features vary simultaneously, the combined data allow some preliminary structure–activity trends to be identified (Figure 4).

The three initially reported pyridine-containing squaramates **3a**–**3c** showed IC_50_ values of 54.53 ± 8.07, 57.81 ± 9.67, and 48.04 ± 7.98 μM, respectively [38]. Compounds **3a** and **3b** contained two-methylene linkers and differed in the position of the pyridine nitrogen, whereas **3c** contained an additional methyl substituent on the linker. The relatively similar activities of **3a** and **3b** suggest that variation in the position of the pyridine nitrogen alone had only a modest effect within this limited series, whereas the somewhat higher activity of **3c** indicates that modification of the linker may also influence DNase I inhibition.

The subsequently investigated chlorine-containing derivatives further demonstrated the sensitivity of inhibitory activity to the overall substituent architecture. The 6-chloropyridin-3-ylmethyl derivative exhibited an IC_50_ value of 43.82 ± 6.51 μM, whereas the related 6-chloro-4-methylpyridin-3-yl derivative lacking a methylene spacer was substantially less active (IC_50_ = 106.60 ± 11.27 μM) [39]. Because these compounds differ in more than one structural feature, the pronounced difference in activity cannot be assigned exclusively to linker length or pyridine substitution. Nevertheless, these observations indicate that the spatial relationship between the pyridine moiety and the squaric acid-derived core is likely to be relevant to DNase I inhibition.

The present series extends this structural framework from monosubstituted derivatives to disubstituted squaramides. Among all nine compounds considered, the symmetric derivative **5a**, bearing two 2-(pyridin-3-yl)ethyl substituents, displayed the highest DNase I inhibitory activity (IC_50_ = 34.42 ± 5.86 μM). In contrast, compounds **5b**–**5d** exhibited relatively similar activities, with IC_50_ values ranging from 53.81 ± 8.88 to 61.95 ± 9.61 μM. Thus, disubstitution of the squaramide core alone does not appear to be sufficient to enhance DNase I inhibition. Rather, the higher activity of **5a** suggests that its particular combination of a symmetric disubstituted squaramide framework and pyridin-3-yl-containing side chains provides a favorable structural arrangement for interaction with DNase I.

Comparison within the current series further indicates that modification of the pyridine-containing substituents can affect inhibitory activity. However, because the compounds differ simultaneously in several structural characteristics, including substituent composition, linker architecture, molecular symmetry, and, in some cases, pyridine chlorination, the contribution of an individual structural feature cannot be unambiguously isolated. Accordingly, the lower activity of the chlorine-containing derivative **5c** relative to **5a** should not be attributed exclusively to the presence of the chlorine atom. Molecular docking and DFT analyses were therefore employed as complementary approaches to explore how the structural and electronic differences among selected compounds may contribute to their interactions with DNase I.

Overall, the combined results for the nine pyridine-containing squaramides and squaramates suggest that DNase I inhibitory activity is influenced by the interplay between the squaric acid-derived core, linker architecture, pyridine substitution pattern, and overall molecular architecture. Within this series, **5a** combines these features in a manner associated with the highest inhibitory activity. Nevertheless, these observations should be regarded as preliminary structure–activity relationships rather than definitive effects of individual structural modifications. A larger series of systematically designed analogues, in which individual structural parameters are varied independently, will be required to establish a more comprehensive SAR.

Molecular docking was therefore employed to investigate whether differences in the binding modes and noncovalent interactions of compounds **5a**–**5d** could provide additional insight into the experimentally observed activity trends.

### 2.8. Molecular Docking Analysis

Molecular docking was performed to predict the preferred binding region of compounds **5a**–**5d** on bovine pancreatic DNase I and to characterize their potential intermolecular interactions. The entire protein surface was examined using 13 docking protocols with different parameters, following the general approach previously applied to pyridine-containing squaramates [38,39]. For each compound, 117 poses were generated, and the nine best poses from each protocol were retained for subsequent analysis.

**Figure 5:**
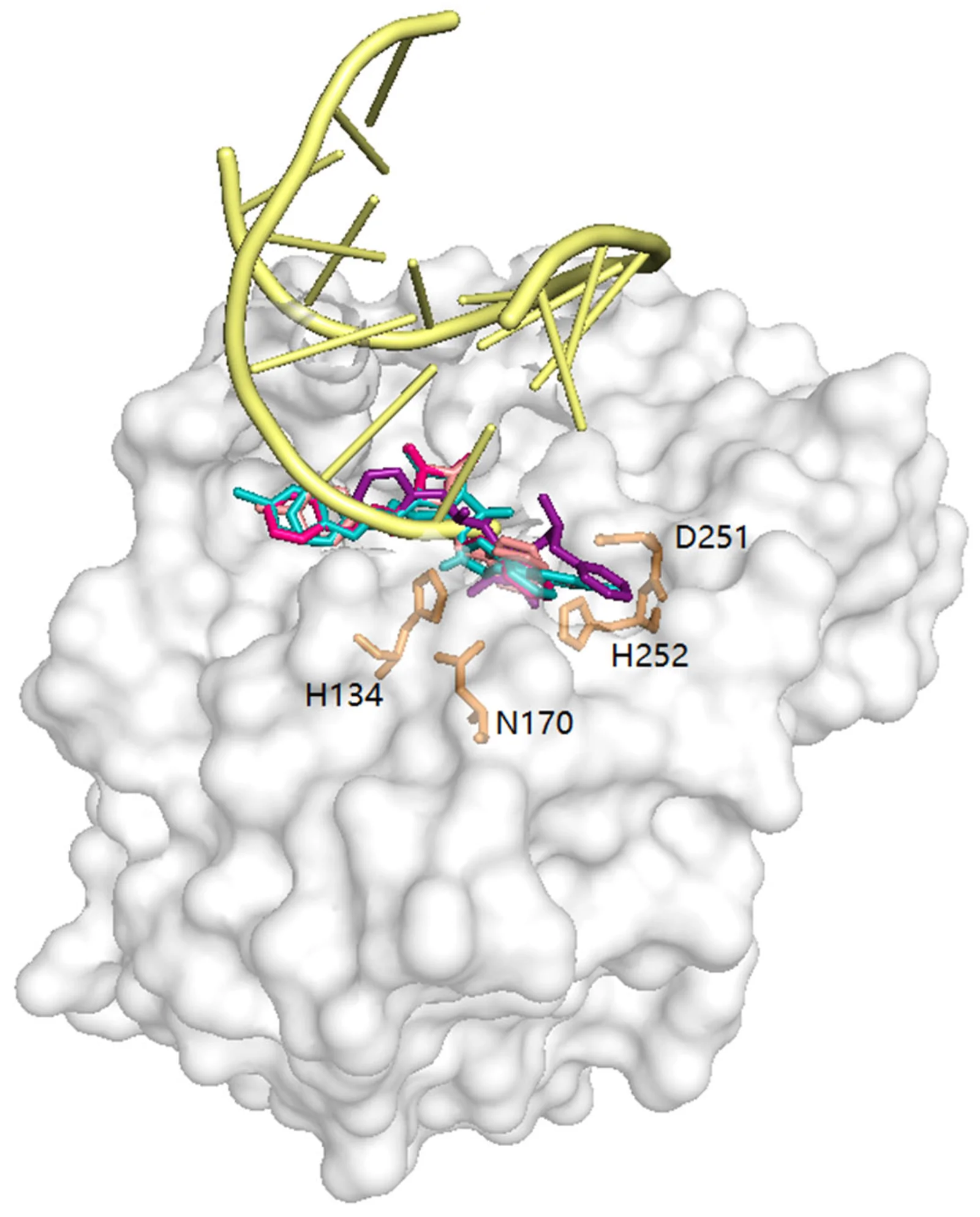
Location of the predicted binding site (BS1) of compounds **5a**–**5d** on the surface of bovine pancreatic DNase I. Compound **5a** is presented in deep pink, **5b** in purple, **5c** in blue, and **5d** in light pink. The crystal structure of the complex between the enzyme and dinucleotide d(GCGATCGC)2 (pdb: 2DNJ [66]) is superimposed. The oligonucleotide is presented in yellow, and some of the amino acid residues of the catalytic center are highlighted in orange.

A common preferred binding site (BS1) was predicted for all four compounds (Figure 5). BS1 accounted for 112 poses (~96%) for **5a**, 113 poses (~97%) for **5c**, and 106 poses (~91%) for both **5b** and **5d**, indicating a strong preference of the compounds for the same region of the DNase I surface. The identified binding site is located within the major oligonucleotide-contact region of the enzyme, in an extensive shallow groove formed between the central β-sheets and lateral loops. The residues defining this region are consistent with those identified in our previous studies and with binding-site predictions and docking analyses reported by other authors [30,32,37,38,39]. They include Asn7, Arg9, Thr10, Glu39, Gly72, Arg73, Asn74, Tyr76, Glu78, Ser110, Arg111, His134, Ala136, Pro137, Asp168, Asn170, Tyr175, Thr203, Thr205, Thr207, Tyr211, Asp251, and His252.

The docking scores of the top-ranked poses ranged from −6.4 to −6.9 kcal/mol. Compounds **5b** and **5c** showed the most favorable predicted affinity (−6.9 kcal/mol), followed by **5d** (−6.8 kcal/mol) and **5a** (−6.6 kcal/mol). Notably, the docking scores did not directly reproduce the experimentally determined inhibitory potency, as **5a** was the most potent inhibitor despite having a less favorable predicted score than **5b** and **5c**. This observation highlights that docking scores should not be interpreted as quantitative predictors of experimental IC_50_ values.

**Figure 6:**
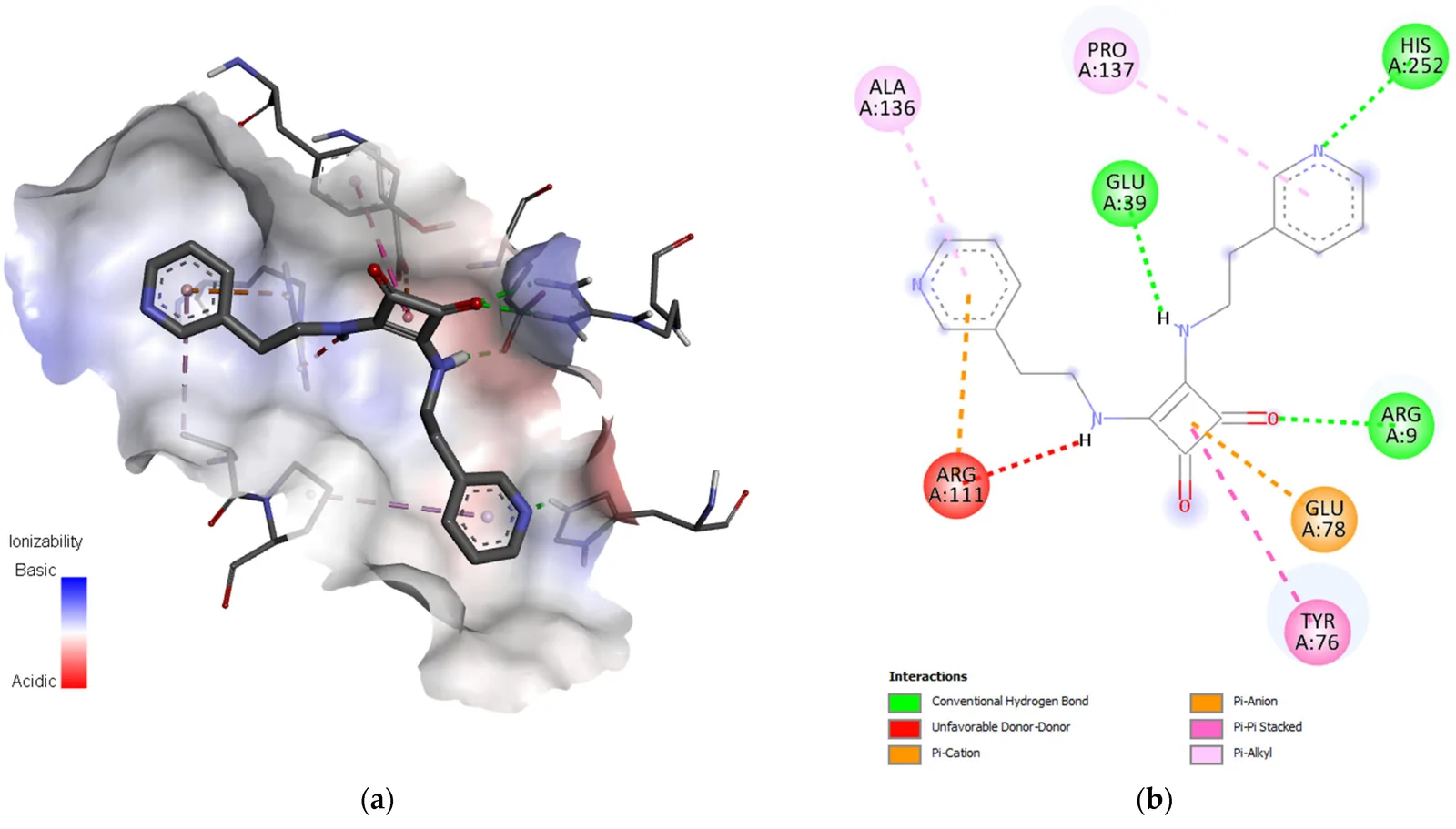
Predicted intermolecular interactions of the top-ranked docking pose of compound **5a** with bovine pancreatic DNase I: spatial view (**a**) and corresponding 2D interaction diagram (**b**). In the 2D interaction diagram, π-cation and π-anion interactions are represented using the same software-generated colour; these interactions can be distinguished by the charged residue involved, with Arg participating in the π-cation interaction and Glu in the π-anion interaction.

The top-ranked docking pose of compound **5a** showed four predicted hydrogen-bonding interactions within BS1 (Figure 6). One squaramide carbonyl oxygen interacts with the guanidino group of Arg9, while the linker amino group forms a hydrogen bond with the Glu39 carboxyl group. The pyridine nitrogen forms a hydrogen bond with the imidazole H-atom of His252 In addition, the pyridine ring participates in a cation–π interaction with the positively charged guanidine group of Arg111 while the squaramide moiety forms an anion–π interaction with Glu78 and a π–π interaction with Tyr76.

**Figure 7:**
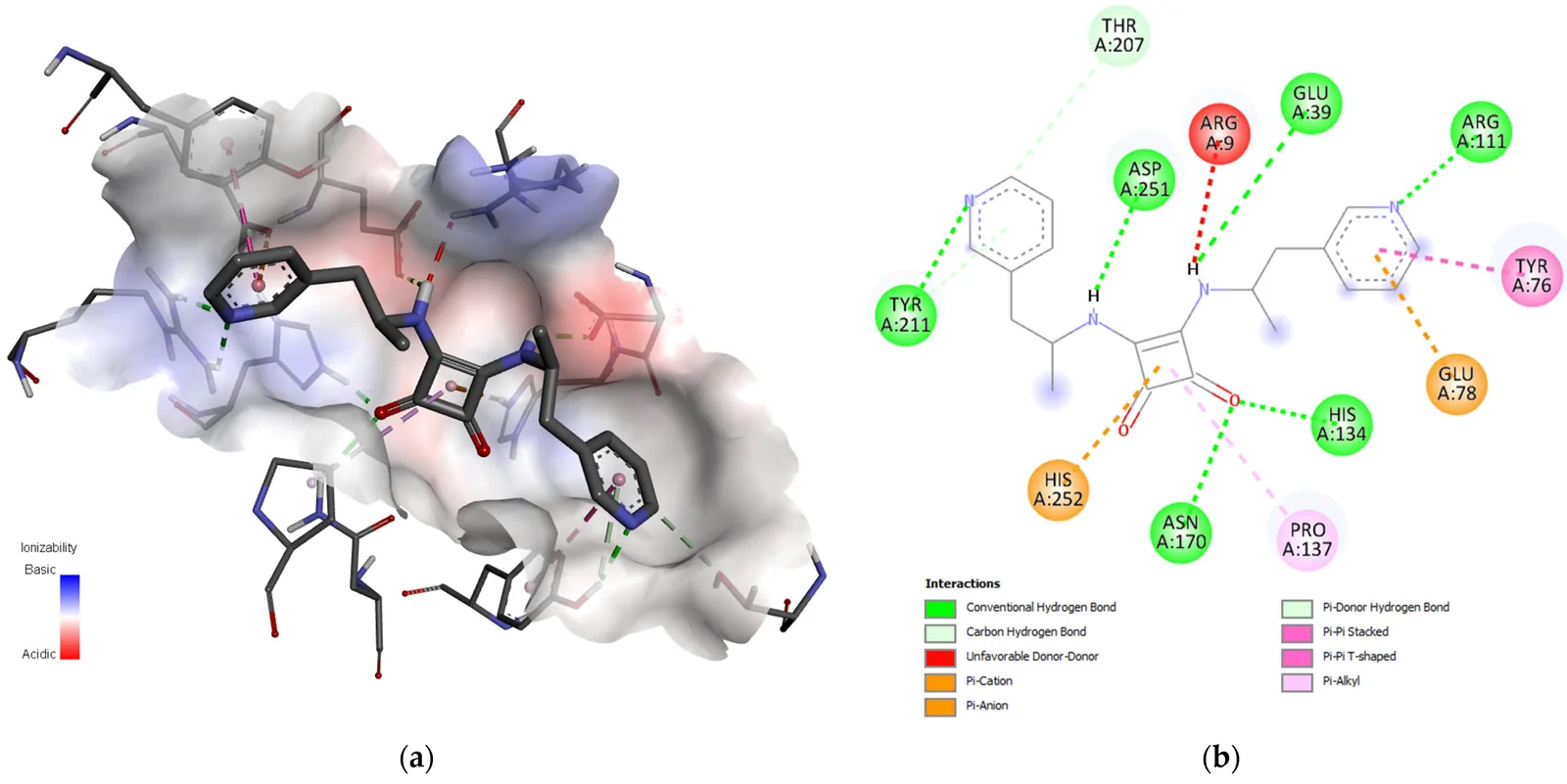
Predicted intermolecular interactions of the top-ranked docking pose of compound **5b** with bovine pancreatic DNase I: spatial view (**a**) and corresponding 2D interaction diagram (**b**). In the 2D interaction diagram, π-cation and π-anion interactions are represented using the same software-generated colour; these interactions can be distinguished by the charged residue involved, with His participating in the π-cation interaction and Glu in the π-anion interaction.

For compound **5b**, several hydrogen-bonding interactions were predicted to stabilize the top-ranked pose (Figure 7). These involve the squaramide carbonyl group, both linker amino groups, and pyridine nitrogen atoms, with the His134 imidazole H-atom, the Asn170 linker amino group, the Glu39 and Asp251 side chain carboxyl groups, the guanidino group of Arg111, and the Tyr211 hydroxyl group. Additional π-mediated interactions were predicted between the pyridine/squaramide moieties and His252, Glu78, and other aromatic residues.

**Figure 8:**
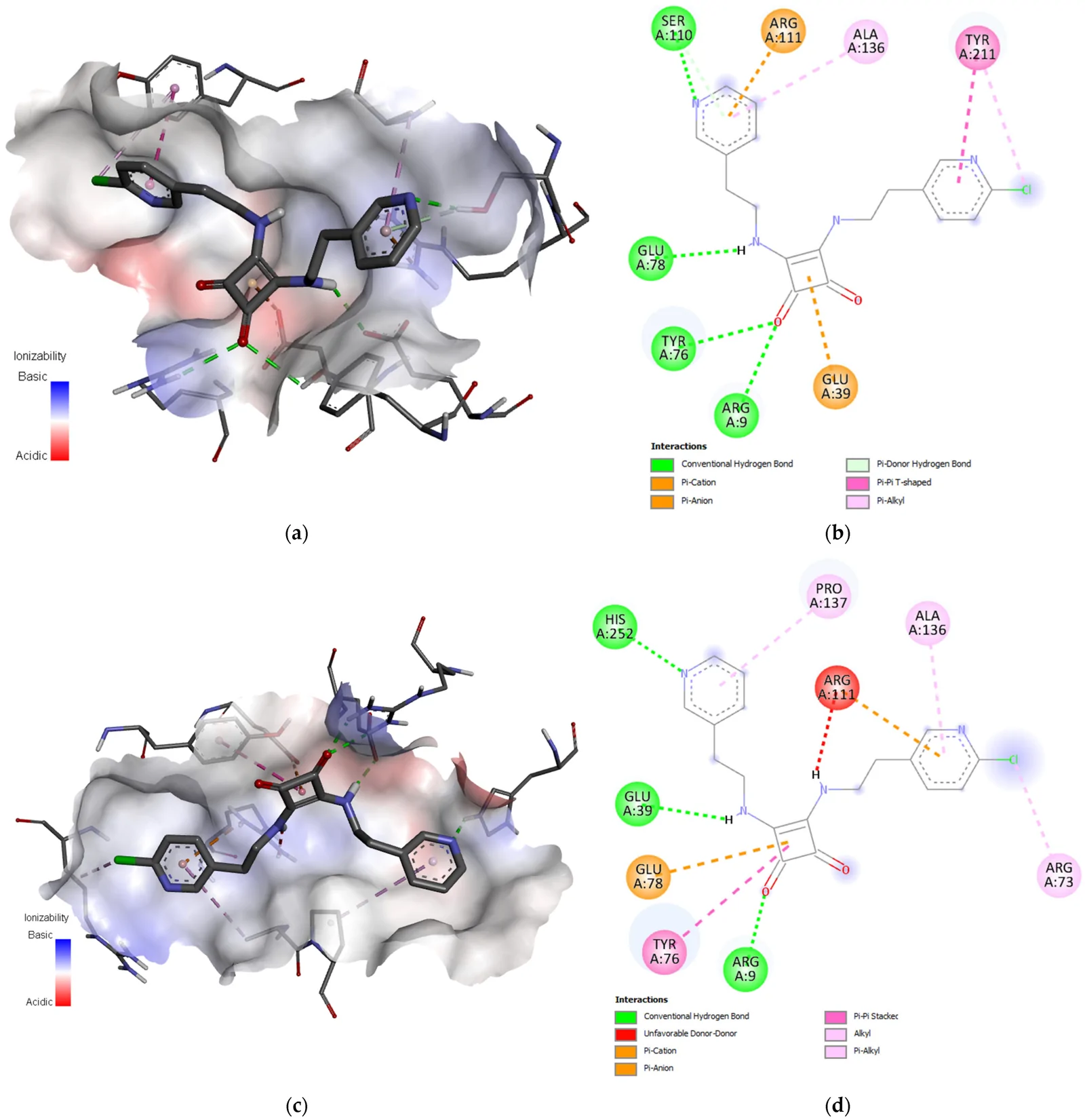
Predicted intermolecular interactions of the two top-ranked docking poses of compound **5c** with bovine pancreatic DNase I: spatial views (**a**,**c**) and corresponding 2D interaction diagrams (**b**,**d**). In the 2D interaction diagram, π-cation and π-anion interactions are represented using the same software-generated color; these interactions can be distinguished by the charged residue involved, with Arg participating in the π-cation interaction and Glu in the π-anion interaction.

The predicted interactions of compound **5c** are illustrated in Figure 8. Four hydrogen bonds were predicted to contribute to stabilization of the docked complex, involving the squaramide carbonyl oxygen, linker amino group, and pyridine nitrogen with the Arg9 guanidino group, the Tyr76 hydroxyl group, the Glu39/Glu78 unprotonated carboxyl group, the Ser110 hydroxyl group, and the His252 imidazole side chain. Additional cation–π, anion–π, and π–π interactions were predicted with Arg111 and the pyridine or 2-Cl-pyridine ring, Glu39 and the squaramide ring, and Tyr211 and the 2-Cl-pyridine moiety, respectively.

**Figure 9:**
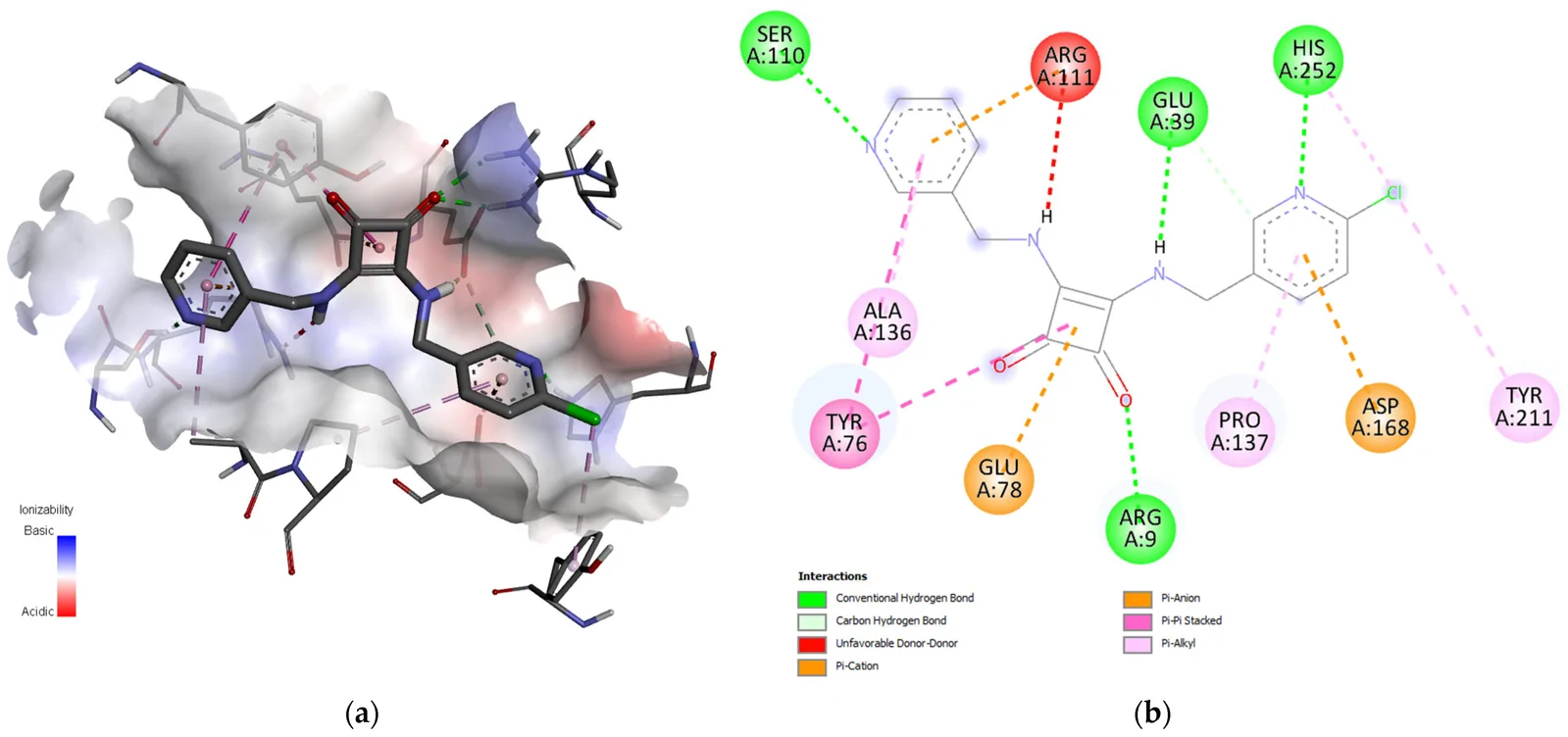
Predicted intermolecular interactions of the top-ranked docking pose of compound **5d** with bovine pancreatic DNase I: spatial view (**a**) and corresponding 2D interaction diagram (**b**). In the 2D interaction diagram, π-cation and π-anion interactions are represented using the same software-generated color; these interactions can be distinguished by the charged residue involved, with Arg participating in the π-cation interaction and Glu and Asp in the π-anion interactions.

Compound **5d** was predicted to be involved in five hydrogen bonds within BS1 (Figure 9), involving the squaramide carbonyl group, both pyridine nitrogen atoms, and the linker amino group with the Arg9 guanidino group, the Ser110 side chain hydroxyl group, the His252 imidazole H-atom, and the Glu39 carboxyl group. Additional cation–π and anion–π interactions were predicted with Arg111, Glu78, and Asp168, while Tyr76 participated in π–π interactions with the pyridine and squaramide rings.

The docking results predicted a common binding mode for the series and identify plausible interactions that may contribute to DNase I inhibition, while the experimentally observed potency differences cannot be explained solely by the calculated docking scores.

Overall, the docking results indicate that all four squaramides may preferentially occupy a common region overlapping with the major DNA-contact surface of DNase I. Several residues involved in the predicted ligand interactions, including Glu39, Glu78, His134, Asn170, Asp251, and His252, have been implicated in the catalytic function of the enzyme, while Arg9, Tyr76, Arg111, and Tyr211 participate in interactions with DNA in the DNase I–oligonucleotide complex. The predicted binding modes suggest a possible structural basis for the experimentally observed DNase I inhibitory activity.

However, molecular docking provides a static and approximate representation of ligand–protein interactions, and the predicted poses and interaction patterns should therefore be interpreted cautiously. The proposed binding region (BS1) has not been experimentally validated and should therefore be regarded as a predicted binding site. In particular, the docking results do not demonstrate direct competition with DNA or establish a specific mechanism of DNase I inhibition. Moreover, the predicted localization of the compounds within the DNA-contact region should not be interpreted as evidence of selectivity for DNase I over other nucleases. Experimental structural, kinetic, binding, and comparative nuclease-inhibition studies will be required to validate the proposed binding region, determine the mode of inhibition, and establish the selectivity profile of these compounds.

Overall, the experimental results identify the pyridine-containing squaramides as DNase I inhibitors, with **5a** exhibiting the highest inhibitory activity within the present series. The molecular docking analysis provides complementary predictions regarding possible binding regions and ligand–protein interactions but does not establish the mechanism of inhibition. These results may serve as a basis for the design and experimental evaluation of further analogues.

### 2.9. Global and Local Molecular Descriptor Analysis

To further investigate the electronic factors that may contribute to the differences in inhibitory activity and binding behavior, compounds **5a** and **5c**, representing the most and least active members of the present series, respectively, were selected for detailed analysis of their global and local molecular reactivity descriptors. The conceptual DFT calculations were performed at the B3LYP/6-311++G(d,p) level of the theory [56,57,58,59,67]. The global molecular descriptors, including the HOMO and LUMO energies, the energy gap between them, ionization potentials, electron affinities, chemical potential, absolute electrophilicity, chemical softness and hardness, and electronegativity index, are presented in Table 4.

**Figure 10:**
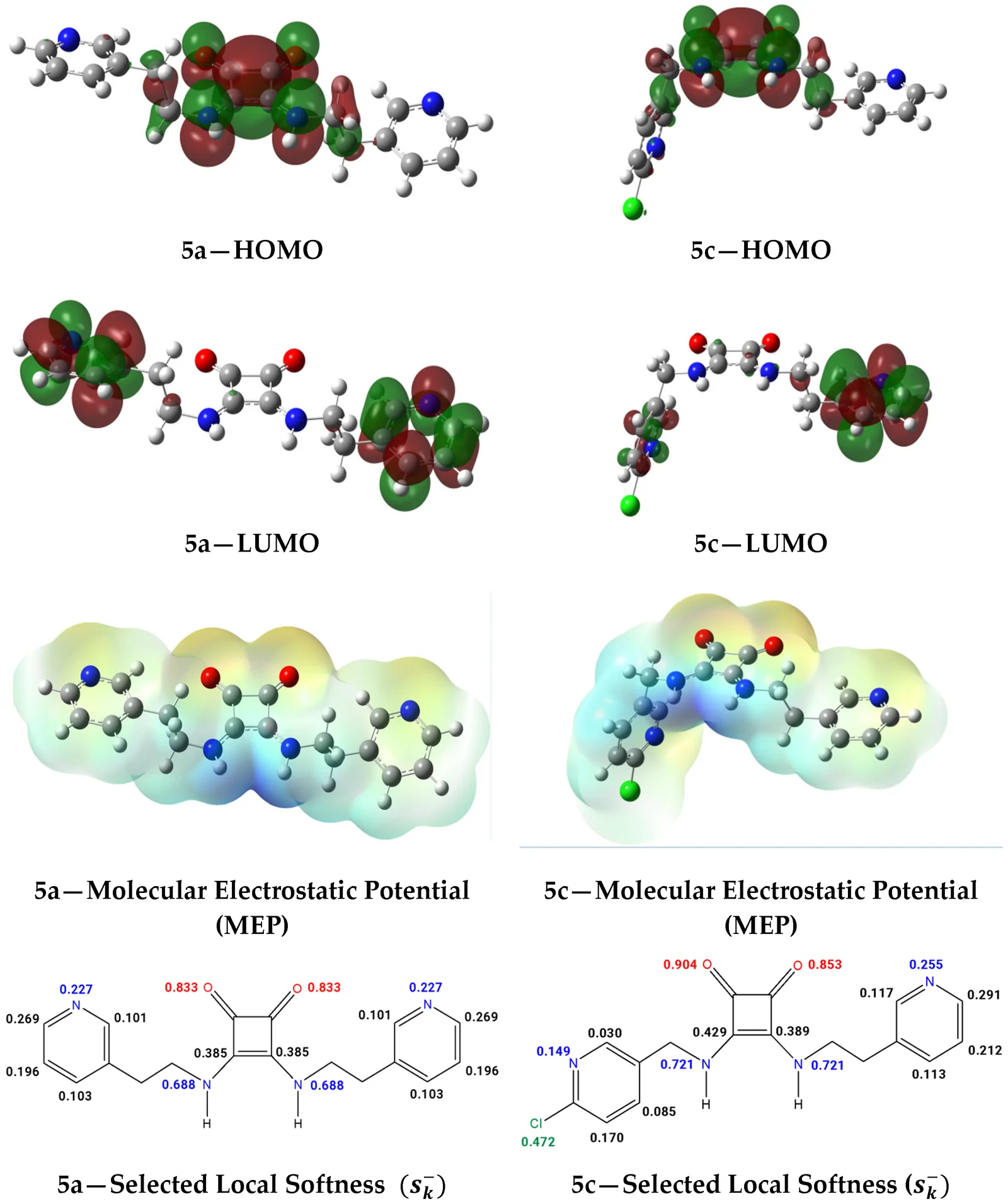
Visualization of selected molecular reactivity descriptors for compounds **5a** and **5c**, including the HOMO and LUMO distributions, molecular electrostatic potential (MEP) maps, and selected local softness $\left(s_{k}^{-}\right)$ distributions.

The calculated descriptors indicate that compound **5a** exhibits a higher ionization potential, greater stability, lower polarizability, and greater overall hardness, which may contribute to an increased propensity for directional electrostatic interactions, including hydrogen bonding, with amino acid residues in the enzyme. As shown in Figure 10, the HOMO is predominantly localized on the squaramide moiety, whereas the LUMO is mainly distributed over the pyridine moieties. The electrostatic potential maps (Figure 10) show a relatively symmetrical potential distribution in compound **5a**, with the most pronounced negative potential localized around the carbonyl oxygen and pyridine nitrogen atoms, which are suitable hydrogen-bond acceptors. In contrast, the amide nitrogen atoms are suitable hydrogen-bond donors.

In compound **5c**, the introduction of the chlorine atom appears to induce an unfavorable spatial distortion and disrupt the symmetry of the electronic distribution, accompanied by a reduction in the electrostatic effect associated with the pyridine moiety. These changes may account for the lower inhibitory activity observed against DNase I. The calculated local softness values are also presented in Figure 9, while the complete set of atomic charges, Fukui indices, and local softness values is provided in the Supplementary Materials (Table S3).

The local softness analysis further indicates that the carbonyl oxygen and pyridine nitrogen atoms are favourable sites for hydrogen-bond acceptance. Although the amide nitrogen atoms and the carbon atoms directly attached to them exhibit relatively high softness values, these values should not be interpreted as indicating a favourable ability to act as hydrogen-bond acceptors, due to the pronounced π–π conjugation within this part of the molecule.

The effect of the substituent in compound **5c** is also evident from the calculated $\left(s_{k}^{-}\right)$ values presented in Table S3. On the one hand, the chlorine substituent disrupts the electronic symmetry of the molecule. On the other hand, it results in increased $\left(s_{k}^{-}\right)$ values across almost the entire molecule and, consequently, a decrease in overall hardness, which may be unfavourable for the formation of strong hydrogen bonds. The third and most pronounced effect is the reduced ability of the pyridine nitrogen atom to participate in hydrogen bonding, resulting from the formation of a highly soft center in its immediate vicinity (Cl, $\left(s_{k}^{-}\right)$ = 0.472).

Overall, the results obtained from the global and local reactivity descriptors are in good agreement with the experimental and molecular docking data, providing further support for the proposed interpretation of the substituent effect on inhibitory activity.

## 3. Materials and Methods

### 3.1. Synthetic Procedure

#### 3.1.1. General Information

All chemical reagents and solvents were of analytical grade and were obtained from Acros Organics (Thermo Fisher Scientific, Waltham, MA, USA) unless otherwise stated. Elemental analyses for carbon, hydrogen, and nitrogen were performed using a EuroEA 3000-CHNS analyzer (Euro Vector S.p.A., Milan, Italy). FT-IR spectra were recorded using a Nicolet iS10 spectrophotometer (Thermo Scientific, Waltham, MA, USA) in the range of 4000–100 cm^−1^ using the attenuated total reflectance (ATR) technique. Spectra were recorded using air as the background, with 100 scans collected at a resolution of 2 cm^−1^.

^1^H and ^13^C NMR spectra were recorded on an Avance NEO 400 spectrometer (Bruker, Ettlingen, Germany) operating at 400 MHz for ^1^H and in DMSO-*d*_6_ as the solvent. Chemical shifts are reported in ppm relative to the residual solvent signal. The following abbreviations were used for signal multiplicities: s, singlet; d, doublet; t, triplet; q, quartet; m, multiplet; dd, doublet of doublets; dq, doublet of quartets; and br, broad signal. Melting points were determined using a BÜCHI 535 melting point apparatus (BÜCHI Labortechnik AG, Meierseggstrasse, Switzerland).

#### 3.1.2. General Procedure for the Synthesis of Symmetric Squaramides:

Diethyl squarate (0.001 mol) and the appropriate pyridine-containing amine (0.002 mol) were dissolved in ethanol, followed by the addition of an excess of triethylamine. The reaction mixture was stirred at room temperature for 48 h. The resulting precipitate was collected by filtration, washed with water, and recrystallized from the appropriate solvent to afford the corresponding symmetric squaramide.

#### 3.1.3. General Procedure for the Synthesis of Asymmetric Squaramides

Diethyl squarate (0.001 mol) and the appropriate first pyridine-containing amine (0.001 mol) were dissolved in ethanol, followed by the addition of an excess of triethylamine. The reaction mixture was stirred at room temperature for 24 h to afford the corresponding monosubstituted squaramate intermediate. Without isolation of the intermediate, an equimolar amount of the second pyridine-containing amine was added to the reaction mixture, and the resulting mixture was stirred continuously until precipitation of the target asymmetric squaramide. The product was collected by filtration and purified by recrystallization.

##### 3,4-bis((2-(Pyridin-3-yl)ethyl)amino)cyclobut-3-ene-1,2-dione **5a**

White crystals; Yield: ca. 84%; Mp: 246.0–248 °C; FTIR (ATR, cm^−1^): 3154, 1797, 1643, 1544, 1477; ^1^H NMR (400 MHz, DMSO *d*_6_, δ, ppm): 8.87 (br t, 1H, NH); 8.67 (br t, 1H, NH); 8.42 (s, 4H, Py); 7.63 (t, 2H, Py); 7.33–7.30 (q, 2H, Py); 4.65–4.53 (dq, 4H, CH_2_); 3.76–3.71 (q, 2H, CH_2_); 3.57–3.51 (m, 2H, CH_2_); ^13^C NMR (101 MHz, DMSO-*d*_6_) δ 189.45, 182.55, 176.98, 173.08, 172.75, 150.54, 150.45, 148.16, 136.96, 136.87, 134.26, 123.91, 69.26, 45.31, 44.79, 34.07, 33.71, 16.05, 16.00. Anal. Calc. for C_18_H_18_N_4_O_2_: C, 67.08; H, 5.59; N, 17.39; Found: C, 67.01; H, 5.48; N, 17.03.

##### 3,4-bis((1-(Pyridin-3-yl)propan-2-yl)amino)cyclobut-3-ene-1,2-dione **5b**

White crystals; Yield: ca. 42%; Mp: 251–256 °C; FTIR (ATR, cm^−1^): 3186, 1787, 1638, 1529, 1480; ^1^H NMR (400 MHz, DMSO-*d*_6_) δ 8.44–8.42 (m, 2H, NH), 8.41 (d, *J* = 2.5 Hz, 2H, Py) 7.59 (dt, *J* = 7.9, 1.9 Hz, 2H, NH), 7.32 (dd, *J* = 7.8, 4.7 Hz, 2H, Py), 7.25 (s, 2H, Py), 1.19 (t, *J* = 6.6 Hz, 6H, CH3); ^13^C NMR (101 MHz, DMSO-*d*_6_) δ 182.42, 167.44, 150.82, 150.77, 148.11, 137.29, 137.22, 134.06, 123.83, 123.80, 51.22, 51.13, 21.58; Anal. Calc. for C_20_H_22_N_4_O_2_: C, 68.56; H, 6.29; N, 16.00; Found: C, 68.81; H, 6.48; N, 16.23.

##### 3-(((6-Chloropyridin-3-yl)methyl)amino)-4-((2-(pyridin-3-yl)ethyl)amino)cyclobut-3-ene-1,2-dione **5c**

White powder; Yield: ca. 78%; Mp: 227.3–232 °C; FTIR (ATR, cm^−1^): 3155, 1797, 1643, 1551, 1475; ^1^H NMR (400 MHz, DMSO *d*_6_, δ, ppm): 8.43 (br t, 2H, NH); 8.37–8.36 (d, 1H, Py); 7.78–7.77 (d, 2H, Py); 7.66–7.64 (d, 2H, Py); 7.54–7.52 (d, 1H, Py); 7.33–7.30 (q, 1H, Py); 4.72–4.71 (d, 2H, CH_2_); 3.77 (s, 2H, CH_2_); 2.88–2.85 (t, 2H, CH_2_); ^13^C NMR (100 MHz, DMSO *d*_6_, δ, ppm): 183.24; 182.96; 168.51; 167.71; 150.37; 149.73; 149.53; 147.99; 139.55; 137.00; 136.86; 134.75; 134.50; 124.78; 123.94; 44.67; 43.95; 34.41; Anal. Calc. for C_17_H_15_N_4_O_2_Cl: C, 59.57; H, 4.41; N, 16.34; Found: C, 59.31; H, 4.59; N, 16.04.

##### 3-(((6-Chloropyridin-3-yl)methyl)amino)-4-((pyridin-3-ylmethyl)amino)cyclobut-3-ene-1,2-dione **5d**

White powder; Yield: ca. 78%; Mp: 224.3–232 °C; FTIR (ATR, cm^−1^): 3154, 1798, 1643, 1553, 1475; ^1^H NMR (400 MHz, DMSO-*d*_6_, 60 °C) δ 8.49 (d, *J* = 2.3 Hz, 1H, Py), 8.45 (dd, *J* = 4.8, 1.7 Hz, 1H, Py), 8.31 (d, *J* = 2.5 Hz, 1H, Py),7.90 (bs, 2H, NH) 7.45 (d, *J* = 8.2 Hz, 1H, Py), 7.39 (ddd, *J* = 7.8, 4.9, 0.9 Hz, 1H, Py), 4.72 (t, *J* = 5.8 Hz, 4H, CH2; ^13^C NMR (101 MHz, DMSO-*d*_6_, 60 °C) δ 183.17, 183.08, 168.33, 168.17, 149.86, 149.25, 148.79, 148.75, 139.49, 136.18, 134.87, 134.33, 124.74, 124.42, 44.98, 44.22; Anal. Calc. for C_16_H_13_N_4_O_2_Cl: C, 58.46; H, 3.96; N, 17.05; Found: C, 58.87; H, 4.46; N, 16.59.

### 3.2. Single-Crystal X-Ray Diffraction (SCXRD)

Single crystals of suitable quality for compounds **5a** and **5b** were mounted on a nylon loop. Diffraction data for both compounds were collected on a Bruker D8 Venture diffractometer equipped (Bruker AXS GmbH, Karlsruhe, Germany) with a PhotonII CMOS detector (Bruker, Karlsruhe, Germany) using microfocus Mo Kα radiation (λ = 0.71073 Å). Data reduction was performed using the APEX4 software [35]. The structures were solved by intrinsic methods using ShelxT and refined by full-matrix least-squares method on F2 with ShelxL program [68]. All non-hydrogen atoms were successfully located from the Fourier map and refined anisotropically. Hydrogen atoms were placed in calculated positions (C–Hmethyl = 0.96 Å, C–Haromatic = 0.93 Å and C–Hmethylenic = 0.97 Å) riding on the parent atom (Ueq = 1.2). Complete crystallographic data for compounds **5a** and **5b** have been deposited in CIF format with the Cambridge Crystallographic Data Center (CCDC) as 2512480 and 2512481 respectively. These data can be obtained free of charge from the CCDC via http://www.ccdc.cam.ac.uk/conts/retrieving.html (accessed on 4 December 2025) or by contacting the CCDC, 12 Union Road, Cambridge CB2 1EZ, UK; Fax: +44-1223336033; E-mail: deposit@ccdc.cam.ac.uk.

### 3.3. Geometry Optimization

Quantum-chemical calculations were performed using the Gaussian 09 software package [69]. The molecular geometries of compounds **5a**–**5d** were optimized using density functional theory (DFT) at the B3LYP/6-311++G(d) level [56,57,58,59]. Geometry optimization was performed without symmetry constraints using an analytical gradient procedure. Harmonic vibrational frequency calculations were subsequently carried out to confirm the nature of the optimized structures as minima on the potential-energy surface. The optimized geometries and calculated structural parameters were used for comparison with the experimental crystallographic data obtained for compounds **5a** and **5b**.

### 3.4. In Silico ADME Prediction

The physicochemical and pharmacokinetic properties of compounds **5a**–**5d** were predicted using the SwissADME web tool (available on https://www.swissadme.ch/index.php, accessed on 9 March 2026) developed by the Swiss Institute of Bioinformatics [70]. The analysis included molecular weight, lipophilicity, hydrogen-bond donors and acceptors, rotatable bonds, topological polar surface area (TPSA), aqueous solubility, gastrointestinal absorption, blood–brain barrier (BBB) permeability, P-glycoprotein substrate status, cytochrome P450 inhibition profiles, oral bioavailability, and synthetic accessibility. The calculated parameters were used to assess the overall drug-likeness and predicted pharmacokinetic profiles of the synthesized compounds.

### 3.5. Pharmacological Assays

#### 3.5.1. DNase I Inhibition Assay

DNase I inhibitory activity was evaluated using bovine pancreatic DNase I by spectrophotometric determination of acid-soluble nucleotides formed during DNA hydrolysis at 260 nm, according to the method of Kolarević et al. [27], with minor modifications. Briefly, the reaction mixture (1040 μL) contained 80 Kunitz units of DNase I from bovine pancreas (type IV, lyophilized powder, ≥2000 Kunitz units/mg protein; Sigma-Aldrich), 0.0077% DNA (sodium salt from calf thymus, type I; Sigma-Aldrich), 80.77 mM Tris-HCl buffer (pH 7.6), and the test compound dissolved in DMSO (final DMSO concentration, 3.85%, *v*/*v*). After incubation at 37 °C for 30 min, the reaction was terminated by addition of 80 μL perchloric acid, and the absorbance was measured at 260 nm. Appropriate solvent and blank controls were included.

Compounds **5a**–**5d** were initially tested at 200 μM. Compounds showing >50% inhibition at this concentration were further evaluated at 50, 100, 150, and 200 μM, and IC_50_ values were determined from concentration–response curves. Crystal violet was used as the reference inhibitor. All measurements were performed in triplicate and averaged.

#### 3.5.2. Cytotoxicicity (MTT Assay)

The cytotoxic potential of compounds **5a**–**5d** was examined in a panel of human cancer cell lines comprising HL-60 (acute myeloid leukemia), MCF-7 (estrogen receptor-positive breast adenocarcinoma), MDA-MB-231 (triple-negative breast adenocarcinoma), and BV-173 (BCR:ABL1-positive leukemia). Cell viability was determined using the MTT assay following the method originally described by Mosmann [71]. All cell lines were obtained from the German Collection of Microorganisms and Cell Cultures (DSMZ, Braunschweig, Germany).

Adherent MDA-MB-231 and MCF-7 cells were maintained as monolayer cultures in RPMI-1640 supplemented with 10% fetal bovine serum (FBS) and non-essential amino acids. Suspension growing cell lines were propagated in RPMI-1640 containing 10% FBS and 2 mM L-glutamine. Cells were incubated at 37 °C under humidified conditions with 5% CO_2_ and were passaged two to three times per week to maintain cultures in the exponential phase of growth.

The cytotoxic activity of the tested compounds was evaluated using the MTT assay, which measures the metabolic conversion of the yellow tetrazolium dye MTT into insoluble purple formazan by metabolically active, viable cells [71]. To account for differences in growth characteristics, suspension and adherent cell lines were seeded at different initial densities. Exponentially growing suspension cells (HL-60 and BV-173) were seeded at 1 × 10^5^ cells/mL (1 × 10^4^ cells/well), whereas adherent cells (MCF-7 and MDA-MB-231) were seeded at the lower density of 1 × 10^4^ cells/mL (1 × 10^3^ cells/well). In both cases, 100 µL of cell suspension was dispensed per well in 96-well plates. Following a 24 h pre-incubation period, the cells were exposed for 72 h to five serial two-fold dilutions of each tested compound, ranging from 400 to 25 µM (400, 200, 100, 50, and 25 µM), with each concentration tested in at least eight replicate wells. At the end of the exposure period, 10 µL of MTT solution (10 mg/mL in PBS) was added to each well, followed by incubation for 4 h at 37 °C. The resulting formazan crystals were solubilized with 100 µL of 5% formic acid in 2-propanol, and absorbance was measured at 580 nm using a Labexim LMR-1 microplate reader (Labexim Products, Vienna, Austria).

Cell viability was expressed as a percentage of the corresponding untreated control, defined as 100%. Concentration-response curves were analyzed by nonlinear regression to determine IC_50_ values, defined as the concentration required to reduce cell viability by 50% relative to the untreated control. Data are presented as mean ± standard deviation (SD) from at least eight replicate determinations. Statistical significance was assessed using Student’s *t*-test, with *p* ≤ 0.05 considered statistically significant.

### 3.6. Molecular Docking

The protein crystal structure was used of bovine pancreatic DNase I in complex with the 5–3 oligonucleotide d(GCGATCGC)_2_ (PDB: 2DNJ; resolution 2 Å) [66] for compounds **5a**–**5d**. The identification of the binding site (BS) was achieved through the utilization of the complete protein surface. AutoDock Vina v.1.2.0 (Center for Computational Structural Biology, CCSB, La Jolla, CA, USA) [72] with the following grid parameters was utilized for the calculations: x-center = 52.369 Å, y-center = 28.297 Å, z-center = 35.826 Å; number of points in x-dimension = 48, y-dimension = 48, z-dimension = 50. In the course of the molecular docking calculations, the protein molecule was kept rigid, whilst the ligand molecules were kept flexible. In order to ensure a thorough conformational search, the exhaustiveness values were set between 8 and 50, and the energy range values were set between 3 and 15. The figures are generated using PyMol v.3.0 (Schrödinger, LLC) [73] and Biovia Discovery Studio v.21.1.0.20298 (San Diego, CA, USA, 2021) [74].

### 3.7. Global and Local Molecular Reactivity Descriptors

The molecular reactivity descriptors of compounds 5a and 5c were calculated at the B3LYP/6-311++G(d,p) level of theory using the energies of the resulting HOMO and LUMO orbitals, in accordance with conceptual density functional theory (CDFT) [56,57,58,59,67]. The global molecular reactivity descriptors were calculated according to the following relationships: ionization potential, (I = −E_HOMO_); electron affinity, (A = −E_LUMO_); chemical potential, (µ = (E_HOMO_ + E_LUMO_)/2); absolute electronegativity, (χ = −µ = (I + A)/2); molecular hardness, (η = (E_LUMO_ − E_HOMO_)/2); molecular softness, (S = ½η); and electrophilicity index, ω_el_ = μ^2^/2η.

Hirshfeld atomic charges [75] were used to calculate the local Fukui indices describing susceptibility toward nucleophilic ($f_{k}^{-}$), electrophilic ($f_{k}^{+}$), and radical ($f_{k}^{0}$) attack [76,77]. The corresponding local softness values were calculated according to $s_{k}^{-}=Sf_{k}^{-}$ [78].

## 4. Conclusions

In the present study, four novel pyridine-containing squaramides (**5a**–**5d**), comprising two symmetric and two asymmetric derivatives, were synthesized and comprehensively characterized by elemental analysis, FT-IR, ^1^H and ^13^C NMR spectroscopy, and, for the symmetric derivatives **5a** and **5b**, single-crystal X-ray diffraction. All four compounds exhibited pronounced inhibitory activity against bovine pancreatic DNase I, with IC_50_ values ranging from 34.42 ± 5.86 to 61.95 ± 9.61 μM. Among the investigated compounds, the symmetric derivative **5a** showed the highest inhibitory activity (IC_50_ = 34.42 ± 5.86 μM), placing it among the most potent synthetic small-molecule DNase I inhibitors reported to date. No measurable cytotoxicity was observed against the investigated human cancer cell lines at the tested concentrations, with cytotoxic effects remaining undetectable even at concentrations exceeding those required for DNase I inhibition.

Comparison of **5a**–**5d** with five structurally related pyridine-containing squaramides and squaramates previously reported by our group provided preliminary insight into the structural features associated with DNase I inhibition. Among the nine compounds considered, 5a exhibited the highest inhibitory activity. However, because several structural parameters vary simultaneously across the series, the contribution of individual structural modifications cannot be unambiguously established, and a larger series of systematically designed analogues will be required to define more comprehensive structure–activity relationships.

Molecular docking predicted a common preferred binding region for compounds **5a**–**5d** within the major DNA-contact/catalytic region of DNase I and identified possible noncovalent ligand-enzyme interactions. However, the docking scores did not reproduce the experimentally observed potency ranking, and the predicted binding modes should be regarded as complementary structural information rather than evidence of a specific inhibition mechanism. The molecular descriptor analysis further characterized electronic differences between selected compounds, while the in silico ADME analysis indicated generally favorable physicochemical and absorption-related properties together with potential P-gp- and CYP-related liabilities requiring experimental evaluation.

Overall, the results identify pyridine-containing squaramides as a useful structural framework for further investigation as DNase I inhibitors, with **5a**, representing a promising compound for subsequent structural optimization. Further studies involving systematically designed analogues, experimental determination of the inhibition mechanism, selectivity assessment, comparative evaluation against other nucleases, and assessment in non-tumor cells and more physiologically relevant models will be required to establish the selectivity and broader biological significance of this compound class.

## Figures and Tables

**Table 1:** Hydrogen-bond geometry for compound **5a**.

D	H	A	d(D–H) (Å)	d(H···A) (Å)	d(D···A) (Å)	∠D–H···A (°)
N1	H1	O2 ^1^	1.00	1.82	2.808(6)	168.2
N4	H4	O3 ^1^	0.88	2.03	2.817(6)	147.9
C8	H8	O2	0.93	2.60	3.459(1)	154.5

^1^ +x, −1 + y, +z.

**Table 2:** Hydrogen-bond geometry for compound **5b**.

D	H	A	d(D–H) (Å)	d(H···A) (Å)	d(D···A) (Å)	∠D–H···A (°)
N1	H1	O2 ^1^	0.86	2.11	2.913(1)	156.3
N4	H4	O3 ^1^	0.86	2.11	2.934(9)	160.2
C12	H12	O3	0.98	2.63	3.265(1)	122.7
C20	H20C	O3 ^1^	0.96	2.60	3.384(1)	138.8
C19	H19C	O2 ^1^	0.96	2.51	3.312(1)	140.9
C5	H5	O2	0.98	2.65	3.298(1)	124.2

^1^ +x, 1 + y, +z.

**Table 3:** Physicochemical, ADME properties and pharmacokinetic parameters of compounds **5a**–**5d** calculated by SwissADME.

Compounds	5a	5b	5c	5d
Formula	C_18_H_18_N_4_O_2_	C_20_H_22_N_4_O_2_	C_17_H_15_ClN_4_O_2_	C_16_H_13_ClN_4_O_2_
Mw, g/mol	322.36	350.41	342.78	328.75
Rot Bonds	8	8	7	6
H-bond Acc	4	4	4	4
H-bond Don	2	2	2	2
Consensus Log P_o/w_	1.35	1.97	1.64	1.41
Drug-likeness *	Yes	Yes	Yes	Yes
Lead-likeness	No; 1 violations: Rotors > 7	No; 2 violations MW > 350, Rotors > 7	Yes	Yes
TPSA, Å^2^	83.98	83.98	83.98	83.98
Log S (ESOL)	−2.8	−3.48	−3.28	−2.98
Solubility, mol/L	1.60 × 10^−3^	3.29 × 10^−4^	5.27 × 10^−4^	1.04 × 10^−3^
Class	Soluble	Soluble	Soluble	Soluble
GI Absorption	High	High	High	High
Oral BA	INSATU	SATU	INSATU	INSATU
BBB permeant	No	No	No	No
P-gp substrate	Yes	Yes	No	No
CYP1A2 inhibitor	Yes	Yes	Yes	Yes
CYP2C19 inhibitor	No	No	Yes	Yes
CYP2C9 inhibitor	No	Yes	Yes	Yes
CYP2D6 inhibitor	No	No	No	No
CYP3A4 inhibitor	Yes	Yes	Yes	Yes
Bioavailability Score	0.55	0.55	0.55	0.55
Synthetic accessibility	3.01	3.97	2.99	2.79

Mw—molecular weight; Rot Bonds—number of rotatable bonds; H-bond Acc—number of hydrogen bond acceptors; H-bond Don—number of hydrogen bond donors; Drug-likeness *—according to all five criteria implemented in SwissADME, Lipinski, Ghose, Veber, Egan, and Muegge; TPSA—topological polar surface area; GI Absorption—gastrointestinal absorption; Oral BA—oral bioavailability; INSATU—insaturation; SATU—saturation; BBB permeant—blood–brain permeation.

**Table 4:** Global molecular reactivity descriptors calculated for compounds **5a** and **5c**.

Descriptor	Symbol	5a	5c
HOMO energy, eV	E_HOMO_	−8.385	−8.355
LUMO energy, eV	E_LUMO_	−5.386	−5.384
HOMO–LUMO energy gap, eV	ΔE_HOMO–LUMO_	3.001	2.971
Ionization potential, kcal mol^−1^	I	193.367	192.683
Electron affinity, kcal mol^−1^	A	124.159	124.109
Chemical potential, a.u.	μ	−0.25301	−0.25242
Absolute electronegativity, a.u.	χ	0.25301	0.25242
Molecular hardness, a.u.	η	0.05515	0.05464
Molecular softness, a.u.^−1^	S	9.0670	9.1508
Electrophilicity index, a.u.	ω_el_	0.58037	0.58305

a.u. atomic units.

## Data Availability

The raw data supporting the conclusions of this article will be made available by the authors on request.

## Supplementary Materials

The following supporting information can be downloaded at: https://www.mdpi.com/article/10.3390/molecules31193548/s1, Table S1. Crystal data and structure refinement for **5a** and **5b**, Table S2. Selected bond lengths (Å), valence and dihedral angles (°) of the studied squaramides **5a**–**5d**; Scheme S1. Numbering of the atoms of the compounds; Figure S1. Optimized molecular structures of **5a**–**5d** calculated by DFT (B3LYP/6-311++G*); IR-ATR of **5a**; IR-ATR of **5b**; IR-ATR of **5c**; IR-ATR of **5d**; ^1^H NMR spectrum of **5a**; ^1^H NMR spectrum of **5b**; ^1^H NMR spectrum of **5c**; ^1^H NMR spectrum of **5d**; ^13^C NMR spectrum of **5a**; ^13^C NMR spectrum of **5b**; ^13^C NMR spectrum of **5c**; ^13^C NMR spectrum of **5d**. Table S3. Calculated atomic Fukui indices and local softness values for compounds **5a** and **5c**.
