# Supplementary material for: Synthesis, Biological Evaluation, and Molecular Docking of Pyridine-Containing Squaramides as DNase I Inhibitors
Source: Molecules. 2026 Oct 5;31(19):3548. doi: 10.3390/molecules31193548 (PMC13667282; doi:10.3390/molecules31193548)
Supplement: Supplementary file 1 [file molecules-31-03548-s001.zip › molecules-4528940-supplementary.pdf]

Synthesis, Biological Evaluation, and Molecular Docking of Pyridine-Containing Squaramides as DNase I Inhibitors

Mariyana Atanasova [1,2,*], Nina Ruseva [3], Georgi Tirolski [4,5], Simeon Stoyanov [4], Ana Marković [6], Andrija Šmelcerović [7], Magdalena Angelova [8], Hristina Sbirkova-Dimitrova [8,9], Adriana Bakalova [1], Rositsa Mihaylova [2,5], and Emiliya Cherneva [1,2,4,*]

[1] Department of Chemistry, Faculty of Pharmacy, Medical University of Sofia, 2 Dunav Str., 1000 Sofia, Bulgaria; a_bakalova@pharmfac.mu-sofia.bg
[2] Centre of Excellence in Informatics and Information and Communication Technologies, Bulgarian Academy of Sciences, Akad. Georgi Bonchev Str., Block 2 and 25A, 1113 Sofia, Bulgaria; rmihaylova@pharmfac.mu-sofia.bg
[3] Department of General and Inorganic Chemistry, University of Chemical Technology and Metallurgy, 1756 Sofia, Bulgaria; nina.ruseva@uctm.edu
[4] Institute of Organic Chemistry with Centre of Phytochemistry, Bulgarian Academy of Sciences, Acad. G. Bontchev Str., Bl. 9, 1113 Sofia, Bulgaria; g_tirolski@abv.bg (G.T.); Simeon.Stoyanov@orgchm.bas.bg (S.S.)
[5] Department of Pharmacology, Pharmacotherapy and Toxicology, Faculty of Pharmacy, Medical University of Sofia, 2 Dunav Str., 1000 Sofia, Bulgaria
[6] Department of Pharmacy, Faculty of Medicine, University of Niš, Blvd. Dr Zorana Đinđića 81, 18000 Niš, Serbia; anule89@yahoo.com
[7] Department of Chemistry, Faculty of Medicine, University of Niš, Blvd. Dr Zorana Đinđića 81, 18000 Niš, Serbia; a.smelcerovic@yahoo.com
[8] Institute of Mineralogy and Crystallography,"Akad. Ivan Kostov", Bulgarian Academy of Sciences, Acad. Georgi Bonchev Str., Bl. 107, 1113 Sofia, Bulgaria; magdalena.angelova@imc.bas.bg (M.A.); hsd@imc.bas.bg (H.S.-D.)
[9] Center of Competence "Personalized Innovative Medicine"—2 PERIMED—2, Vasil Aprilov Blvd. 15A, 4002 Plovdiv, Bulgaria
* Correspondence: matanasova@pharmfac.mu-sofia.bg (M.A.); cherneva@pharmfac.mu-sofia.bg (E.C.)

Table S1. Crystal data and structure refinement for **5a** and **5b.**

Identification code	**5a**	**5b**
Empirical formula	$C_{18}H_{18}N_4O_2$	$C_{22}H_{22}N_2O_2$
Formula weight	322.36	350.41
Temperature/K	290	290
Crystal system	Orthorhombic	Orthorhombic
Space group	$Pna2_1$	$Pca2_1$
a/Å	12.7397(1)	14.1607(2)
b/Å	6.0679(6)	6.1809(5)
c/Å	20.9136(2)	20.1840(2)
α/°	90	90
β/°	90	90
γ/°	90	90
Volume/Å^3	1616.7(3)	1766.7(3)
Z	4	4
ρ_{calc}g/cm^3	1.324	1.310
μ/mm^{-1}	0.089	0.084
F(000)	680.0	744.0
Crystal size/mm^3	0.25×0.2×0.15	0.15×0.1×0.05
Radiation	Mo $K\alpha$ (λ = 0.71073)	Mo $K\alpha$ (λ = 0.71073)
2Θ range for data collection/°	6.396 to 52.782	4.036 to 50.052
Reflections collected	17561	22585
R_{int} /R_{sigma}	0.1009/0.0679	0.1025/0.0818
Data/restraints/parameters	2982/1/217	3102/37/237
Goodness-of-fit on F^2	1.151	1.128
Final R indexes [I>=2σ (I)]	R_1 = 0.0843 wR_2 = 0.1457	R_1 = 0.1242 wR_2 = 0.3051
Final R indexes [all data]	R_1 = 0.1126 wR_2 = 0.1556	R_1 = 0.1322 wR_2 = 0.3148
Largest diff. peak/hole / e Å^{-3}	0.21/-0.21	1.04/-0.51
Flack parameter	-2.4(10)	0.2(10)
CCDC number	2512480	2512481

Table S2. Selected bond lengths (Å), valence and dihedral angles (o) of the studied squaramides **5a–5d**.

Geometric parameters	Exp./B3LYP/6-311++G*				
Bond lengths	5a		5b	5c	5d
	Exp.	B3LYP/6-311++G*	Exp.	B3LYP/6-311++G*	B3LYP/6-311++G*
C1-C2	1.46	1.48	1.48	1.48	1.48
C1-C4	1.42	1.39	1.40	1.39	1.39
C2-O6	1.22	1.20	1.21	1.20	1.20
C3-O5	1.21	1.21	1.21	1.21	1.21
C3-C2	1.49	1.53	1.53	1.53	1.53
C1-N7	1.30	1.34	1.34	1.34	1.34
C4-N11	1.32	1.34	1.34	1.34	1.34
N7-C8	1.45	1.46	1.47	1.46	1.47
N11-C12	1.46	1.47	1.47	1.47	1.47
Valence angles					
C1C2O6	137.21	135.06	135.84	134.77	132.41
C1N7C8	120.50	122.14	125.84	122.11	122.19
C2C1N7	133.51	132.74	135.50	132.34	132.41
C3C4N11	134.69	132.74	135.50	132.42	132.50
C4N11C12	120.51	122.14	125.84	122.52	122.67
C4C1N7	133.60	134.54	132.04	134.87	134.83
N11C4C1	135.44	134.53	132.04	135.01	134.91
Dihedral angles					
C1C4N11C12	179.74	175.57	179.05	174.59	173.21
C4C1N7C8	-179.45	175.58	179.05	-172.00	-172.99
O5C3C4N11	5.09	0.93	0.13	1.56	2.06
C2C1N7C8	-2.84	-6.75	-1.54	10.65	10.07

C3C4N11C12	-1.84	-6.76	-1.55	-7.30	-9.44
N7C8C9C10	162.08	-179.45	-167.73	-85.05	-86.87
N11C12C13C14	169.34	-179.46	-167.74	-179.34	84.65

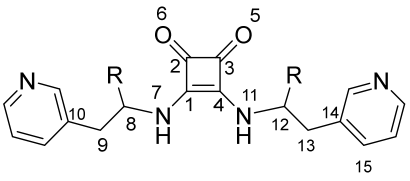

5a R=H
5b R=CH_3

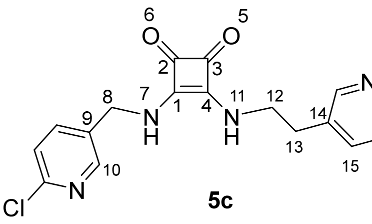

5c

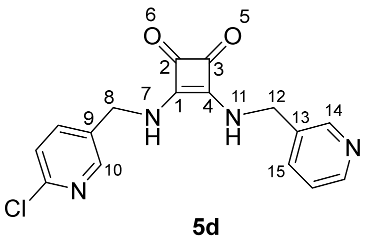

5d

Scheme S1. Numbering of the atoms of the compounds.

Figure S1. Optimized molecular structures of **5a–5d** calculated by DFT (B3LYP/6-311++G*).

5a

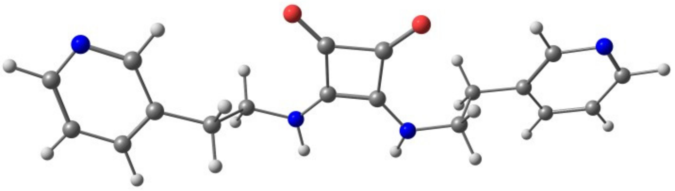

5b

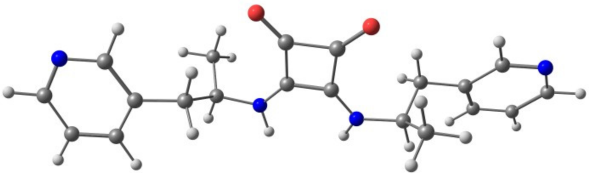

5c

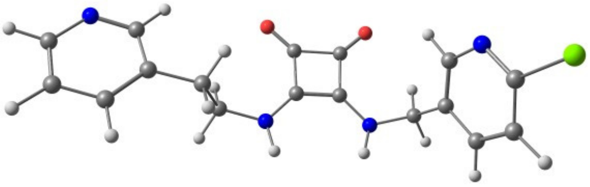

5d

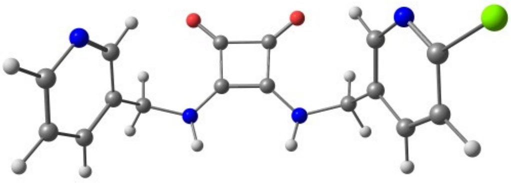

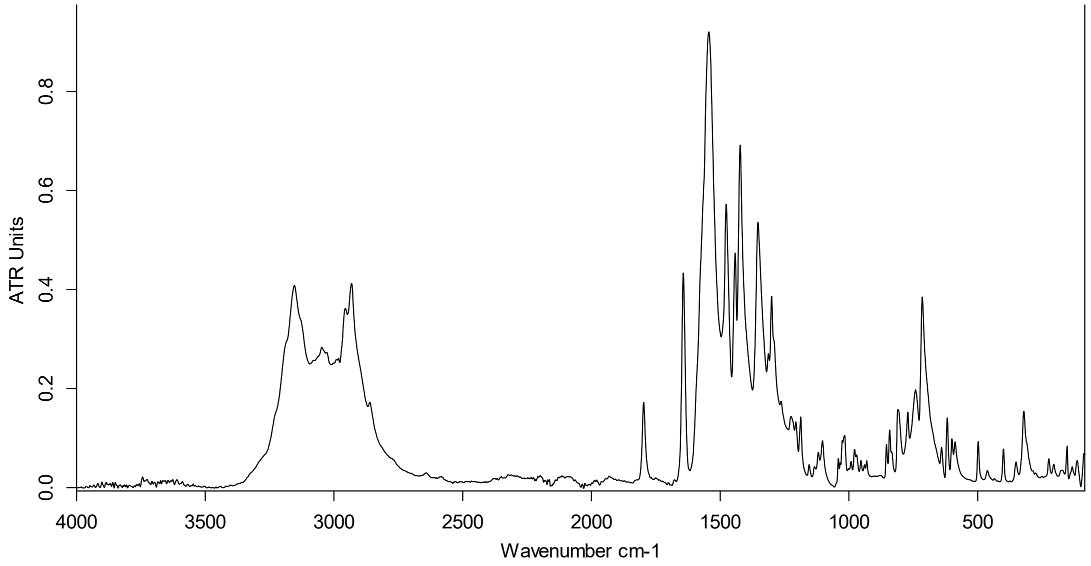

IR-ATR of **5a**

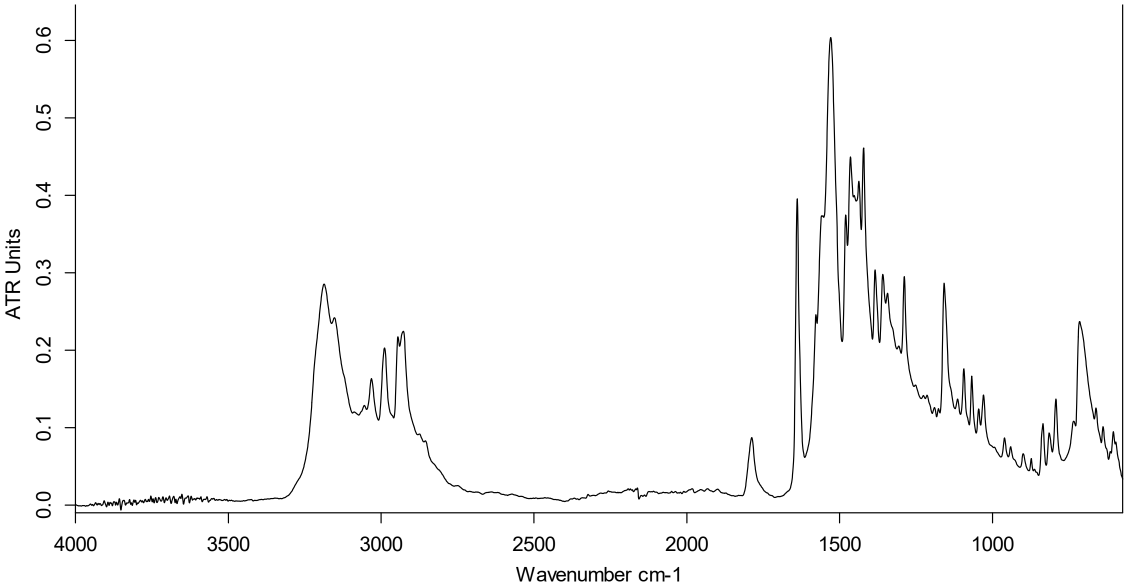

IR-ATR of **5b**

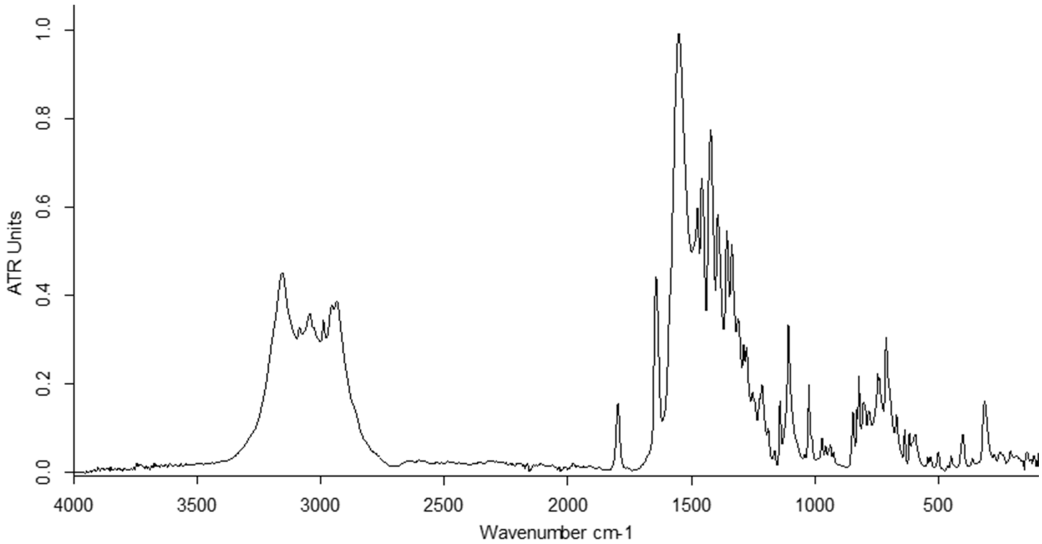

IR-ATR of **5c**

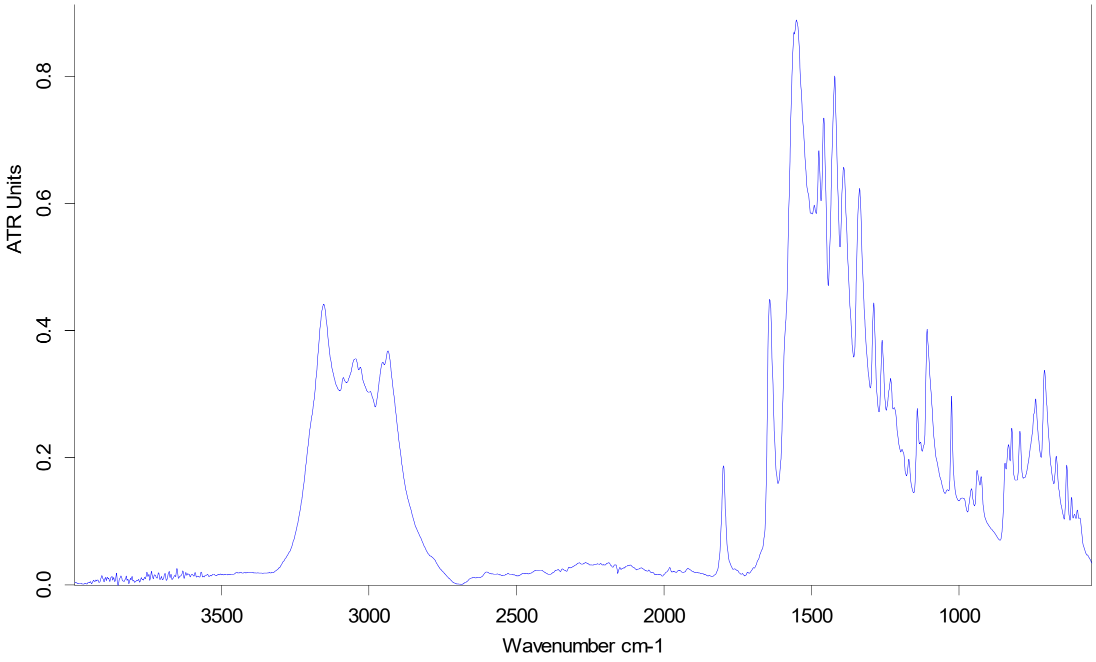

IR-ATR of **5d**

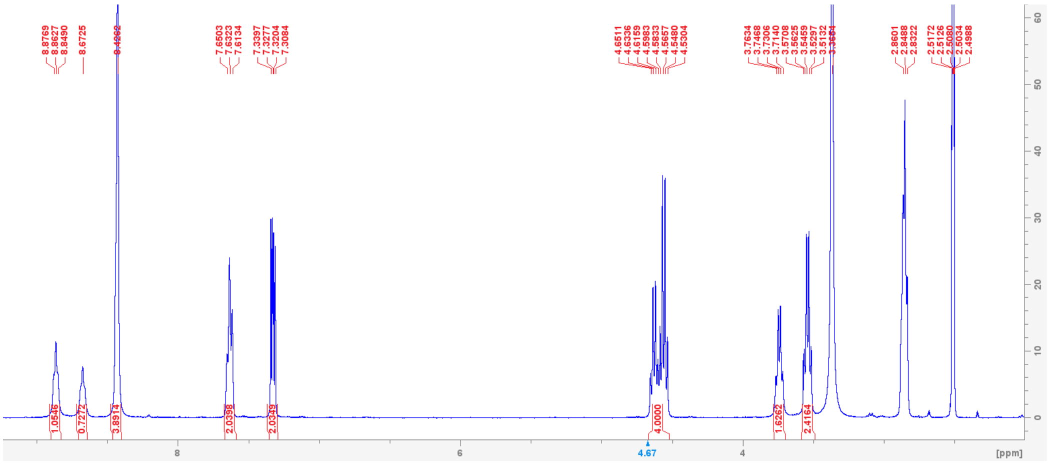

^{1}H NMR spectrum of **5a**

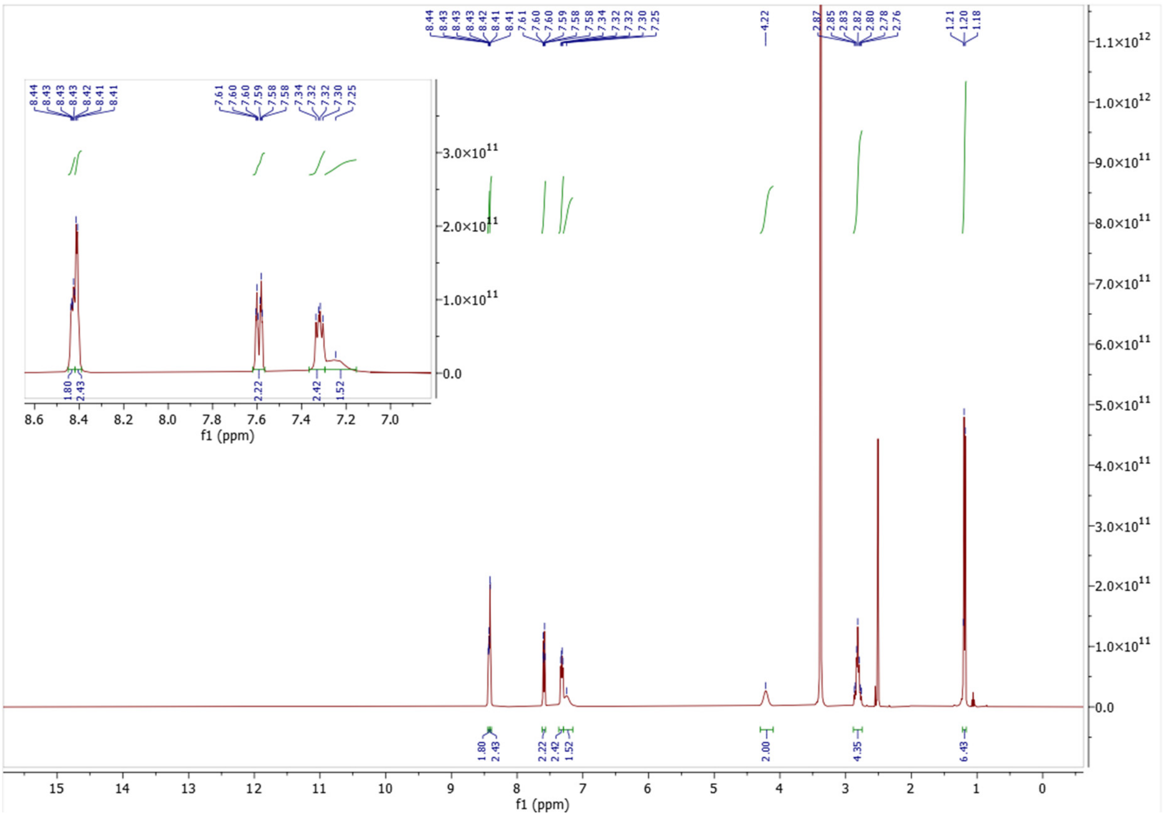

^{1}H NMR spectrum of **5b**

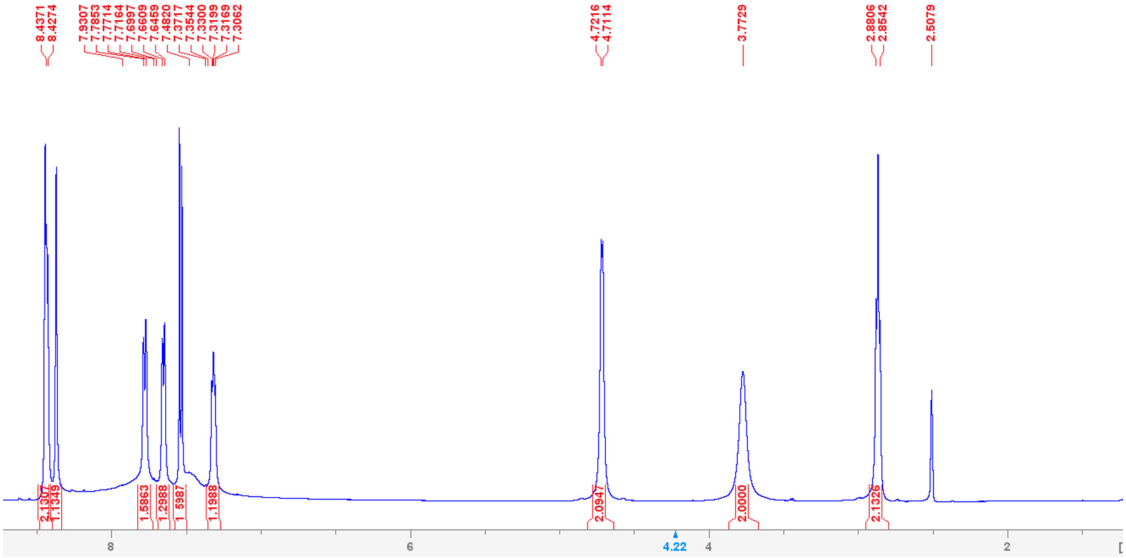

^{1}H NMR spectrum of **5c**

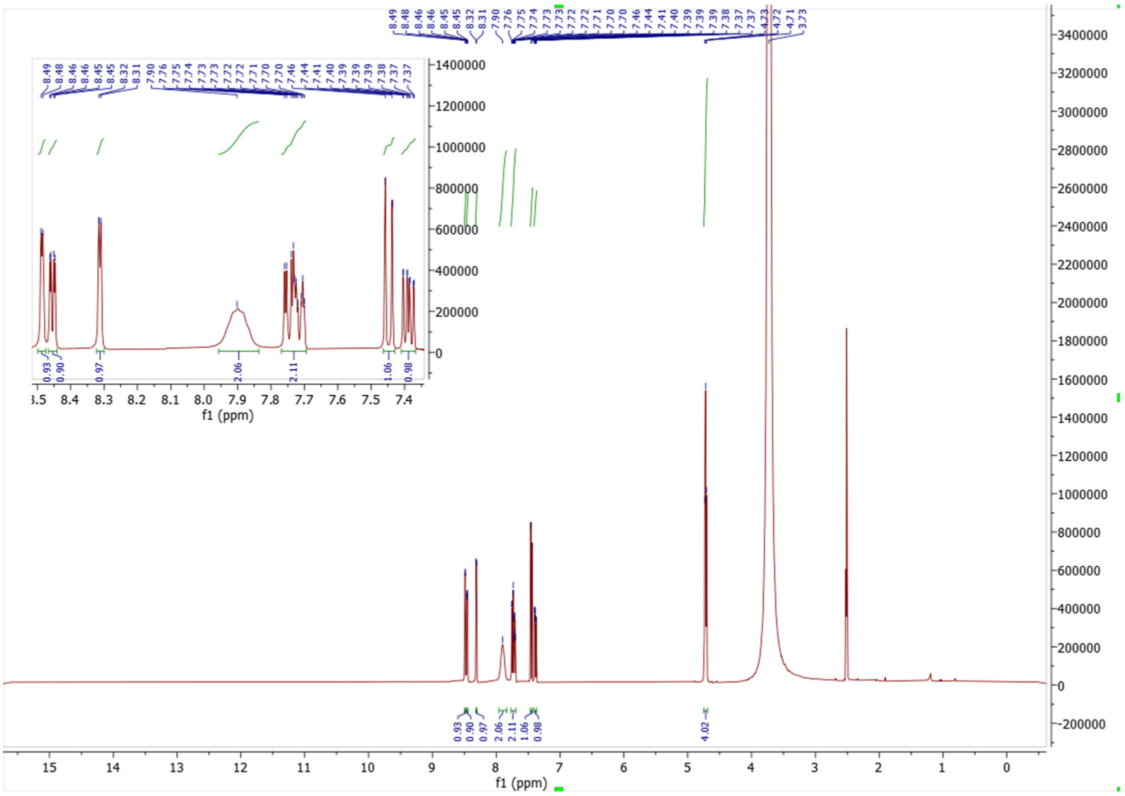

^{1}H NMR spectrum of **5d**

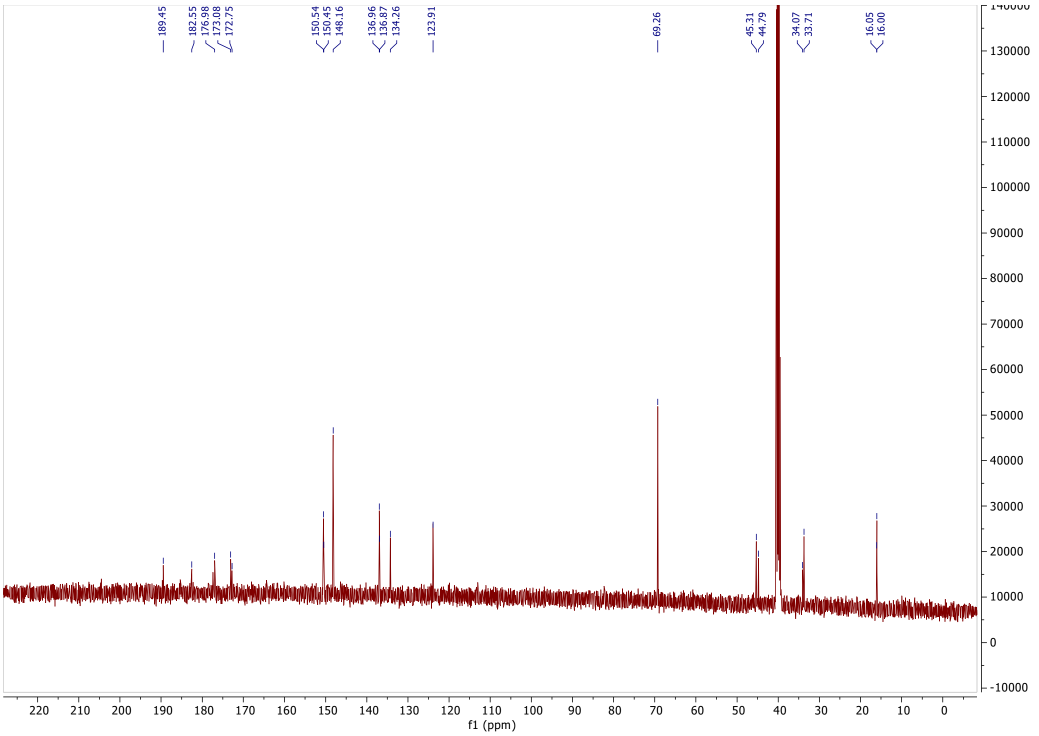

^{13}C NMR spectrum of **5a**

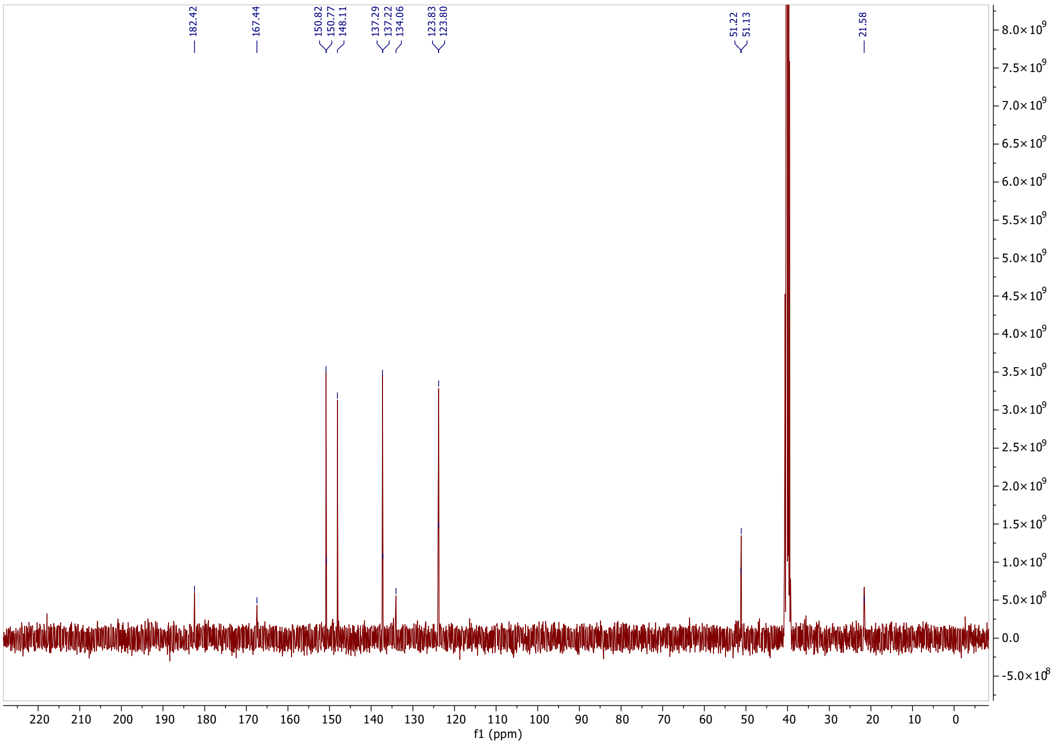

^{13}C NMR spectrum of **5b**

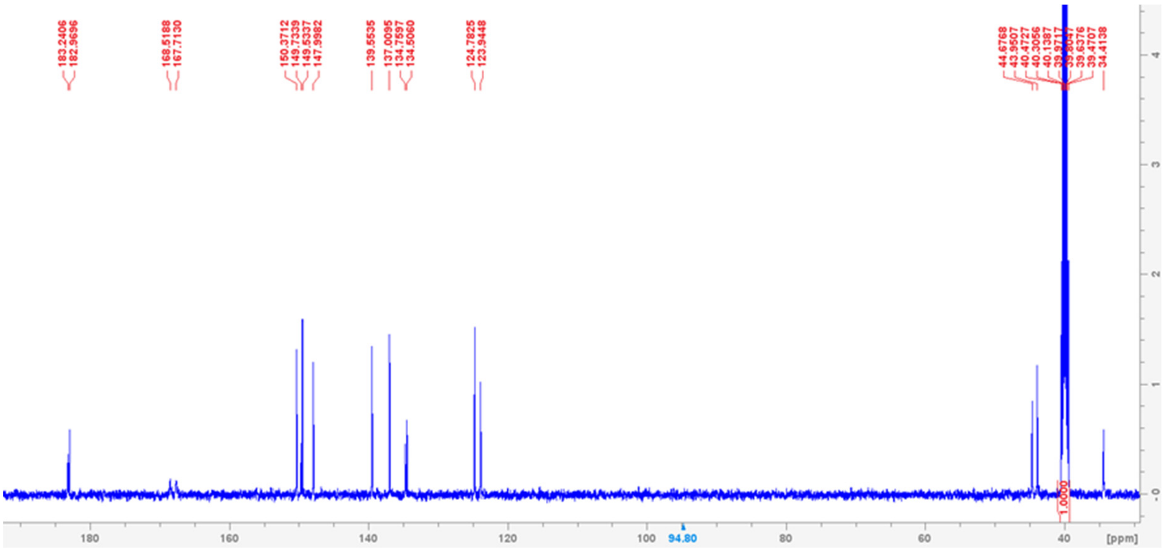

^{13}C NMR spectrum of **5c**

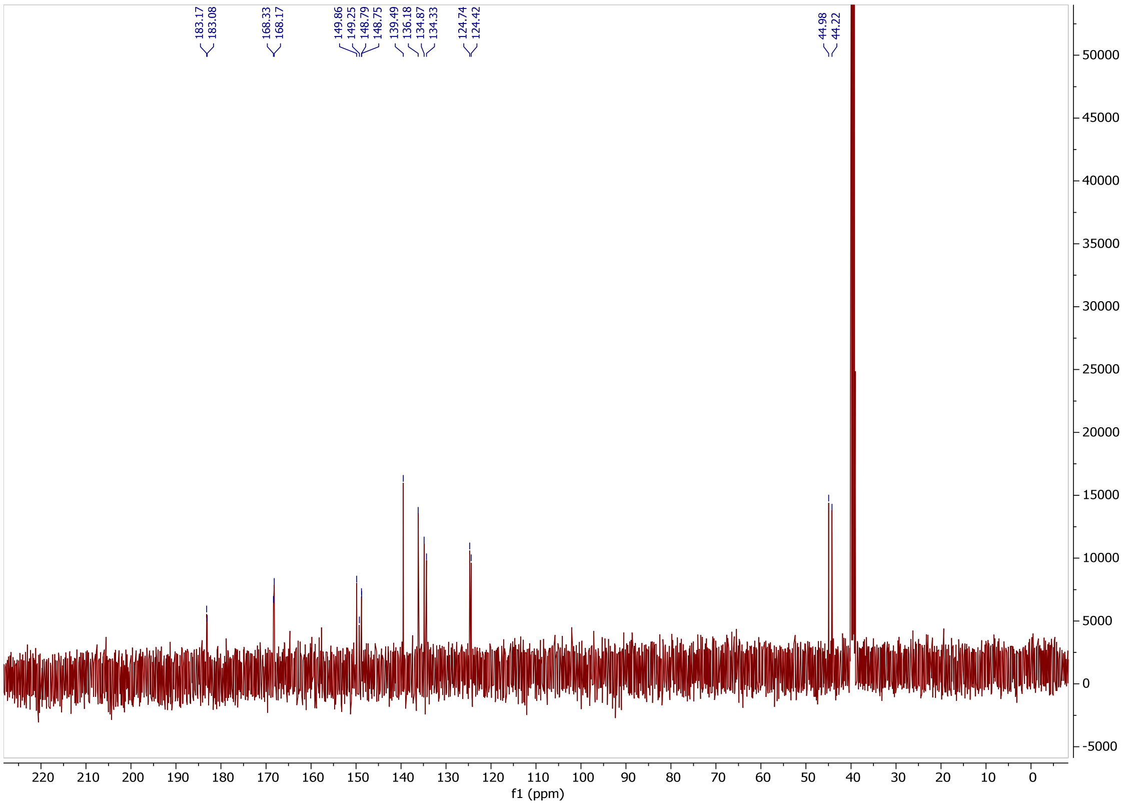

^{13}C NMR of **5d**

Table S3. Calculated atomic Fukui indices and local softness values for compounds **5a** and **5c**.

5a

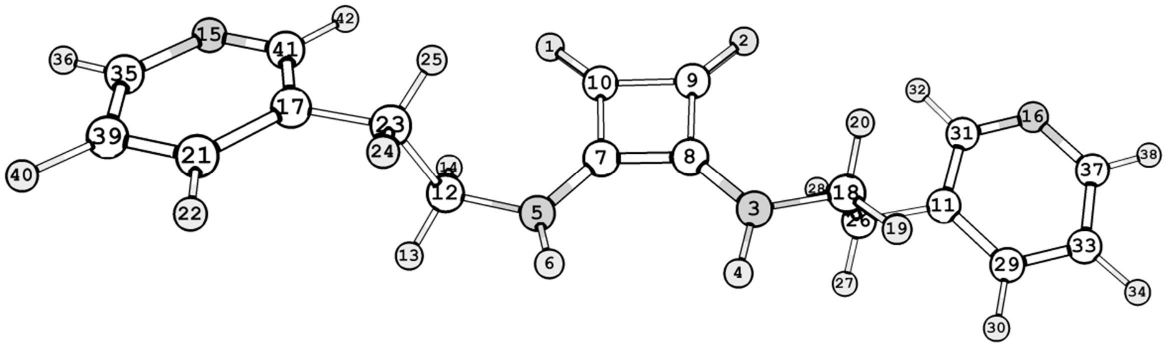

No	Atom	Charge$_{M}$	Charge$_{RA}$	Charge$_{RK}$	f_k^+	f_k^-	f_k^0	s_k^+	s_k^-	s_k^0
1	O	-0.274	-0.304	-0.183	0.030	0.092	0.076	0.273	0.831	0.688
2	O	-0.274	-0.304	-0.183	0.030	0.092	0.076	0.273	0.831	0.688
3	N	-0.078	-0.094	-0.003	0.016	0.076	0.054	0.145	0.688	0.489
4	H	0.121	-0.029	0.154	0.150	0.033	0.166	1.361	0.296	1.509
5	N	-0.078	-0.094	-0.003	0.016	0.076	0.054	0.145	0.688	0.489
6	H	0.121	-0.029	0.154	0.150	0.033	0.166	1.361	0.296	1.509
7	C	0.030	0.017	0.073	0.014	0.043	0.035	0.124	0.385	0.316
8	C	0.030	0.017	0.073	0.014	0.043	0.035	0.124	0.385	0.316
9	C	0.105	0.089	0.142	0.016	0.037	0.035	0.146	0.338	0.315
10	C	0.105	0.089	0.142	0.016	0.037	0.035	0.146	0.338	0.315
11	C	0.000	-0.005	0.003	0.005	0.003	0.006	0.042	0.032	0.058
12	C	0.008	-0.013	0.021	0.021	0.013	0.027	0.189	0.121	0.249
13	H	0.037	0.003	0.058	0.034	0.021	0.045	0.311	0.191	0.406
14	H	0.045	0.035	0.057	0.010	0.012	0.016	0.088	0.110	0.143
15	N	-0.158	-0.188	-0.133	0.029	0.025	0.042	0.265	0.227	0.378
16	N	-0.158	-0.188	-0.133	0.029	0.025	0.042	0.265	0.227	0.378
17	C	0.000	-0.005	0.003	0.005	0.003	0.006	0.042	0.032	0.058
18	C	0.008	-0.013	0.021	0.021	0.013	0.027	0.189	0.121	0.249

19	H	0.037	0.003	0.058	0.034	0.021	0.045	0.311	0.191	0.406
20	H	0.045	0.035	0.057	0.010	0.012	0.016	0.088	0.110	0.143
21	C	-0.029	-0.053	-0.018	0.023	0.011	0.029	0.212	0.103	0.264
22	H	0.043	0.021	0.051	0.021	0.009	0.026	0.192	0.080	0.232
23	C	-0.050	-0.060	-0.039	0.010	0.011	0.015	0.091	0.097	0.139
24	H	0.029	0.009	0.040	0.020	0.011	0.026	0.184	0.102	0.235
25	H	0.039	0.033	0.042	0.006	0.004	0.008	0.056	0.032	0.072
26	C	-0.050	-0.060	-0.039	0.010	0.011	0.015	0.091	0.097	0.139
27	H	0.029	0.009	0.040	0.020	0.011	0.026	0.184	0.102	0.235
28	H	0.039	0.033	0.042	0.006	0.004	0.008	0.056	0.032	0.072
29	C	-0.029	-0.053	-0.018	0.023	0.011	0.029	0.212	0.103	0.264
30	H	0.043	0.021	0.051	0.021	0.009	0.026	0.192	0.080	0.233
31	C	0.023	0.009	0.034	0.013	0.011	0.019	0.119	0.101	0.169
32	H	0.048	0.041	0.051	0.007	0.003	0.009	0.065	0.028	0.079
33	C	-0.045	-0.064	-0.023	0.019	0.022	0.030	0.176	0.196	0.274
34	H	0.046	0.031	0.061	0.015	0.016	0.022	0.132	0.142	0.203
35	C	0.018	-0.006	0.048	0.024	0.030	0.039	0.215	0.269	0.349
36	H	0.043	0.027	0.061	0.016	0.018	0.025	0.147	0.164	0.229
37	C	0.018	-0.006	0.048	0.024	0.030	0.039	0.215	0.269	0.349
38	H	0.043	0.027	0.061	0.016	0.018	0.025	0.147	0.164	0.229
39	C	-0.045	-0.064	-0.023	0.019	0.022	0.030	0.176	0.196	0.274
40	H	0.046	0.031	0.061	0.015	0.016	0.022	0.132	0.142	0.203
41	C	0.023	0.009	0.034	0.013	0.011	0.019	0.119	0.101	0.169
42	H	0.048	0.041	0.051	0.007	0.003	0.009	0.065	0.028	0.079
**5c**										

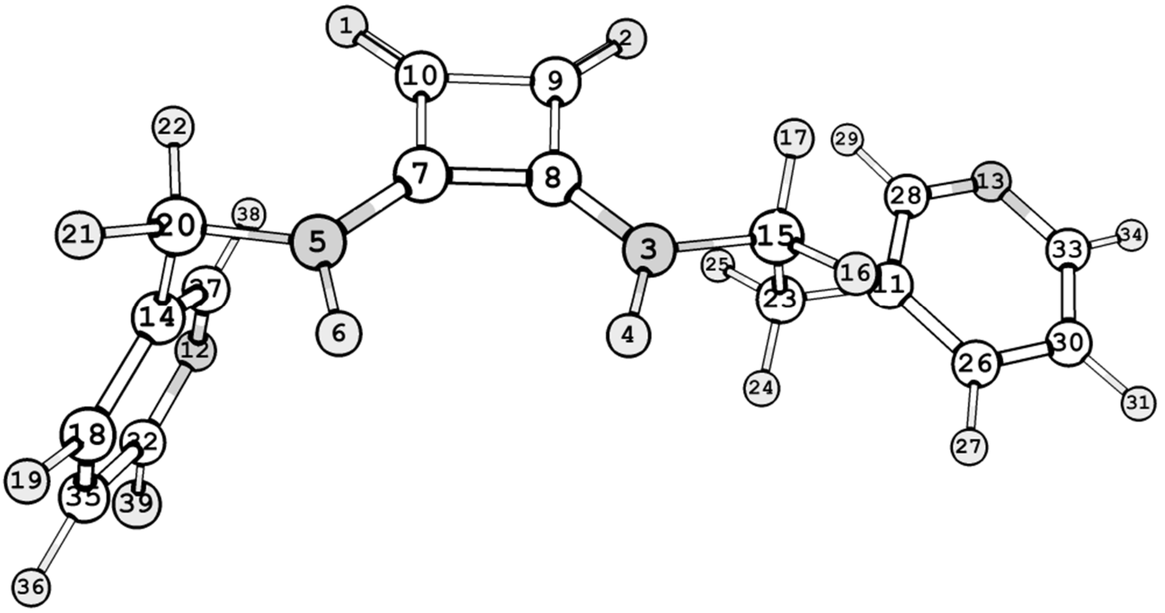

No	Atom	ChargeM	ChargeRA	ChargeRK	f_k^+	f_k^-	f_k^0	s_k^+	s_k^-	s_k^0
1	O	-0.273	-0.300	-0.174	0.027	0.099	0.076	0.247	0.904	0.699
2	O	-0.272	-0.304	-0.179	0.032	0.093	0.079	0.295	0.853	0.722
3	N	-0.074	-0.091	0.005	0.016	0.079	0.056	0.149	0.721	0.509
4	H	0.123	-0.017	0.156	0.140	0.034	0.157	1.284	0.307	1.437
5	N	-0.080	-0.093	-0.001	0.013	0.079	0.052	0.120	0.721	0.480
6	H	0.120	0.013	0.152	0.107	0.032	0.123	0.979	0.290	1.124
7	C	0.029	0.019	0.075	0.010	0.047	0.033	0.089	0.426	0.301
8	C	0.033	0.022	0.076	0.011	0.042	0.033	0.105	0.389	0.299
9	C	0.107	0.090	0.144	0.017	0.038	0.036	0.155	0.345	0.327
10	C	0.105	0.091	0.144	0.014	0.040	0.034	0.127	0.363	0.309
11	C	0.000	-0.002	0.005	0.002	0.005	0.004	0.017	0.044	0.039
12	N	-0.148	-0.186	-0.132	0.037	0.016	0.046	0.342	0.149	0.417
13	N	-0.158	-0.183	-0.130	0.025	0.028	0.039	0.232	0.255	0.359
14	C	-0.012	-0.031	-0.015	0.019	-0.003	0.018	0.175	-0.026	0.162
15	C	0.009	-0.010	0.023	0.019	0.014	0.026	0.173	0.129	0.238

16	H	0.038	0.006	0.060	0.032	0.022	0.043	0.296	0.200	0.396
17	H	0.045	0.036	0.058	0.009	0.013	0.016	0.086	0.115	0.143
18	C	-0.023	-0.069	-0.014	0.046	0.009	0.051	0.423	0.085	0.466
19	H	0.048	0.007	0.057	0.041	0.009	0.046	0.375	0.083	0.417
20	C	0.009	-0.012	0.022	0.022	0.013	0.028	0.197	0.119	0.256
21	H	0.042	0.008	0.064	0.034	0.022	0.045	0.314	0.201	0.415
22	H	0.052	0.039	0.061	0.013	0.009	0.017	0.118	0.084	0.160
23	C	-0.049	-0.058	-0.038	0.008	0.011	0.014	0.076	0.104	0.128
24	H	0.029	0.012	0.041	0.017	0.012	0.023	0.157	0.106	0.210
25	H	0.040	0.035	0.044	0.004	0.004	0.006	0.040	0.038	0.059
26	C	-0.029	-0.048	-0.017	0.019	0.012	0.025	0.171	0.113	0.227
27	H	0.043	0.025	0.052	0.018	0.010	0.022	0.161	0.088	0.204
28	C	0.023	0.011	0.035	0.012	0.013	0.018	0.107	0.117	0.166
29	H	0.048	0.042	0.052	0.006	0.004	0.008	0.054	0.037	0.072
30	C	-0.044	-0.062	-0.021	0.018	0.023	0.029	0.163	0.212	0.269
31	H	0.046	0.033	0.062	0.013	0.017	0.022	0.121	0.152	0.197
32	C	0.081	0.047	0.098	0.034	0.018	0.042	0.308	0.161	0.388
33	C	0.019	-0.002	0.051	0.020	0.032	0.036	0.187	0.291	0.332
34	H	0.043	0.029	0.063	0.014	0.019	0.024	0.132	0.177	0.221
35	C	-0.045	-0.069	-0.026	0.024	0.019	0.033	0.219	0.170	0.304
36	H	0.052	0.033	0.066	0.019	0.014	0.026	0.175	0.132	0.240
37	C	0.034	0.018	0.037	0.016	0.003	0.018	0.148	0.030	0.162
38	H	0.051	0.039	0.050	0.011	0.000	0.011	0.101	-0.003	0.100
39	Cl	-0.059	-0.117	-0.007	0.058	0.052	0.084	0.533	0.472	0.769
